# *Lmx1a* Encodes a Rostral Set of Mesodiencephalic Dopaminergic Neurons Marked by the *Wnt*/B-Catenin Signaling Activator *R-spondin 2*

**DOI:** 10.1371/journal.pone.0074049

**Published:** 2013-09-16

**Authors:** Elisa J. Hoekstra, Lars von Oerthel, Lars P. van der Heide, Willemieke M. Kouwenhoven, Jesse V. Veenvliet, Iris Wever, Yong-Ri Jin, Jeong K. Yoon, Annemarie J. A. van der Linden, Frank C. P. Holstege, Marian J. Groot Koerkamp, Marten P. Smidt

**Affiliations:** 1 Swammerdam Institute for Life Sciences, University of Amsterdam, Amsterdam, The Netherlands; 2 Neuroscience and Pharmacology, Rudolf Magnus Institute of Neuroscience, University Medical Center Utrecht, Utrecht, The Netherlands; 3 Molecular Cancer Research, University Medical Center Utrecht, Utrecht, The Netherlands; 4 Center for Molecular Medicine, Maine Medical Center Research Institute, Maine, United States of America; Radboud University, Netherlands

## Abstract

Recent developments in molecular programming of mesodiencephalic dopaminergic (mdDA) neurons have led to the identification of many transcription factors playing a role in mdDA specification. LIM homeodomain transcription factor *Lmx1a* is essential for chick mdDA development, and for the efficient differentiation of ES-cells towards a dopaminergic phenotype. In this study, we aimed towards a more detailed understanding of the subtle phenotype in *Lmx1a*-deficient (dreher) mice, by means of gene expression profiling. Transcriptome analysis was performed, to elucidate the exact molecular programming underlying the neuronal deficits after loss of *Lmx1a*. Subsequent expression analysis on brain sections, confirmed that *Nurr1* is regulated by *Lmx1a,* and additional downstream targets were identified, like *Pou4f1, Pbx1, Pitx2*, *C130021l20Rik*, *Calb2* and *Rspo2*. In line with a specific, rostral-lateral (prosomer 2/3) loss of expression of most of these genes during development, *Nurr1* and *C130021l20Rik* were affected in the SNc of the mature mdDA system. Interestingly, this deficit was marked by the complete loss of the *Wnt*/b-catenin signaling activator *Rspo2* in this domain. Subsequent analysis of *Rspo2−/−* embryos revealed affected mdDA neurons, partially phenocopying the *Lmx1a* mutant. To conclude, our study revealed that *Lmx1a* is essential for a rostral-lateral subset of the mdDA neuronal field, where it might serve a critical function in modulating proliferation and differentiation of mdDA progenitors through the regulation of the *Wnt* activator *Rspo2*.

## Introduction

Mesodiencephalic dopaminergic (mdDA) neurons function in regulating motor control and emotion related behavior, and loss of these neurons can cause Parkinson’s disease, and other neurological disorders as well. In order to understand the molecular mechanisms causing the selective degeneration of these neurons, insight in the pathways and factors involved in the development and maintenance of this subset of dopaminergic neurons is needed.

The onset of mdDA development is induced by several extrinsic factors such as *Shh*, *Fgf8* and *Wnt1* [1]–[3]. In addition to this early signaling from the organizing centers in the developing CNS, several developmental factors are essential for the subdivision into different domains, and for the differentiation of newborn neurons towards mdDA neurons [3]. Among the factors implicated in mdDA neuron development, are *Wnt1*, *Wnt5a*, *En1/2*, *Otx2*, *Foxa1*, *Foxa2*, *Ngn2*, *Nurr1* (*Nr4a2*), *Pitx3*, *Msx1*, and the LIM homeodomain transcription factors *Lmx1a* and *Lmx1b* [4]–[14].

Several studies suggested a role for *Lmx1a* in establishing the mdDA neuronal phenotype [5], [15]. Gain-and-loss-of-function studies in chick revealed that *Lmx1a* is needed for the specification of mdDA neurons. Moreover, *Lmx1a* can induce mouse embryonic stem cells (ESCs) into DA neurons [5]. In addition, a *Wnt1-Lmx1a* auto-regulatory loop was identified when differentiating ES cells into mdDA neurons [15]. Together, these experiments, in chick and ESCs, suggest an essential role for *Lmx1a* in the determination of mdDA neurons. Contradictory, studies performed in *Lmx1a* mutant mice, revealed a subtle phenotype only, with mild reduction of *Th* and *Nurr1*, and compromised *Wnt*-expression [13]. Other studies in *Lmx1a* mutants also revealed a moderate reduction in a number of (VTA) neurons [16], [17]. Besides several studies indicating that *Lmx1a* functions in proliferation and neurogenesis, the precise role of *Lmx1a* in the mouse mdDA neuronal system is still not fully understood.

Therefore, to understand the *Lmx1a* phenotype in depth, we studied the loss of function of *Lmx1a* in the *Dreher* mouse, which can be considered as a null mutant [17]–[19]. From this, we established that *Lmx1a* is essential for a rostral-lateral part of the developing mdDA system. Furthermore, to elucidate the molecular programming causing this phenotype, we performed microarray analysis on dissected brain material of *Lmx1a-dr/dr* embryos. This revealed several *Lmx1a* target genes, such as *Nurr1*, *C130021L20Rik* and *Rspo2*. The loss of these genes was region specific, and occurred in a rostral group of mdDA neurons. Subsequent analysis in *Rspo2−/−* embryos revealed a subtly affected mdDA neuronal field, partially resembling the *Lmx1a* phenotype. Altogether, *Lmx1a* is essential for the correct programming of a rostral subset of developing mdDA neurons, marked by the *Wnt*/b-catenin signaling activator *Rspo2*.

## Materials and Methods

### Animals

The *Lmx1a* mutant (strain name B6C3Fe a/a-*Lmx1a* ^dr-J^/J) was obtained from Jackson Laboratory (Bar Harbor, ME), and maintained on a C57Bl/6J background (Charles River). Genotyping was done by PCR using primers: forward 5′-CAAAGAGCCCCTGGAG and reverse 5′-GCATACGGATGGACTTCCC, resulting in a 236 bp fragment containing the drJ mutation. Due to this drJ point mutation, a HpyCH4V-restriction site disappears. Wild-type PCR product is restricted into fragments of 52, 88 and 95 bp. *Lmx1a-dr/dr* product results in 140 and 95 bp fragments. For embryo generation, heterozygous *Lmx1a* mice were crossed, and the day of detection of a copulatory plug was considered E0.5. Used animals were euthanized by CO2 asphyxiation, or decapitation. *Rspo2-LacZ* mutant mice were generously provided by Jeong Kyo Yoon and Yong-Ri Jin [20]. Mice were maintained under standard conditions and all efforts were made to minimize suffering. All procedures were according to and fully approved by the Dutch Ethical Committee for animal experimentation of the University Medical Center Utrecht (DEC UMC-U, The Netherlands), and the University of Amsterdam (UvA, The Netherlands).

### In Situ Hybridization

Embryos and postnatal brains were frozen on dry ice. Sections (16 um) were cut and collected on SuperFrost+ slides (Menzel-Glaser). In situ hybridization (ISH) with digoxigenin-labeled RNA probes was performed as described previously [21], [22]. The following probes were used: *Th,* a 1142 bp fragment of rat cDNA [23]; *Aadc*, fragment containing bp 22–488 of the mouse coding sequence [22]; *NurrI*, the 3′-region of rat *NurrI* transcript; *Ahd2*, fragment containing bp 568–1392 of the coding sequence; *En1*, the 5′-region of the transcript; *Lmx1a*, an 1150 bp fragment containing bp 218–1366 of the coding sequence; *Pitx3*, a 285 bp fragment of the 5′-region of rat *Pitx3* transcript [22]. For microarray post ISH-analysis, cDNA from RNA originating from E14.5 mouse midbrains was used for PCR with gene specific primers (Table S1). The PCR fragments were cloned into pGEM-t-easy and sequenced. Probes were generated by means of T7 or SP6 RNA DIG-labeling according to manufacturer’s protocol (Roche).

### Immunohistochemistry

Embryos were incubated in 4% para-formaldehyde in PBS at 4°C, followed by cryoprotection in 30% sucrose in PBS, before freezing on dry ice. For immunohistochemistry, sections were washed twice for 5 min in TBS, blocked in 4% fetal calf serum (FCS) or 5% normal donkey serum in TBS for 30 min, and were washed again. Slides were incubated with primary antibody in THZT (50 mM Tris-HCl pH 7.6, 0.5 M NaCl, 0.5% Triton) overnight, washed 3× with TBS for 5 min and incubated 1 h with secondary antibody. Slides were washed three times 10 min in PBS and mounted using FluorSave (Calbiochem). Antibodies used: rabbit anti-TH (Pel-Freez, 1∶1000), sheep anti-TH (Millipore, 1∶1000), rabbit anti-LMX1A (a kind gift of M. German, UCSF, 1∶1000), rabbit anti-PITX3 [9](1∶500) and AHD2 (Abcam, 1∶100). Secondary antibodies: goat anti-rabbit Alexa-Fluor-488 and -555, donkey anti-sheep Alexa-Fluor-488, all 1∶1000 (Invitrogen).

### Combined In Situ Hybridization/Immunohistochemistry

ISH on fresh frozen sections was performed as described [21], [22]. After this, slides were washed in TBS, incubated in 0.3% H2O2 in TBS for 30 min, washed again, blocked with 4% FCS in TBS for 30 min, washed again and incubated overnight with rabbit anti-TH (Pel-Freez, 1∶1000) in TBST (0.05 M Tris-HCl pH 7.4, 0.9% NaCl, 0.5% Triton). Next day, sections were washed in TBS, incubated for 1 h with avidin-biotin-peroxidase reagent mix (ABC Elite kit, Vector Laboratories) in TBST. Slides were washed again, and stained with 3,3′-diamino-benzidine (DAB) until TH regions showed a clear staining (with maximum of 10 min staining). The reaction was ended through washing with water, slides were dehydrated with ethanol and mounted using Entellan (Merck).

### qPCR

Analysis was performed on a LightCycler 480 II (Roche) using a One Step SYBR green kit (Qiagen) according to the manufacturer’s protocol. Dissected midbrain total RNA (10 ng) was used as a template. Water was used as a non-template control. All samples were normalized to 18 s reference. Table S2 lists primer sets used for qPCR.

### Microarray Analysis

RNA was isolated from E12.5 dissected midbrains of Lmx1a-dr/dr and Lmx1a+/+ embryos, using Trizol according to manufacturer’s protocol (Invitrogen). Each experimental sample consisted of RNA derived from 5 midbrains, pooled and purified on a column, according to protocol (Qiagen, RNeasy mini kit). All RNA samples were analyzed using a 2100 BioAnalyzer (Agilent Technologies) to ensure the quality of the RNA. Microarray analysis was performed on 4 experimental samples, hybridized to a reference pool of RNA derived from 20 Lmx1a+/+ midbrains. Microarray analysis was performed as described with slight modifications [24]. Agilent Mouse Whole Genome Gene Expression Microarrays V1 (Agilent Technologies, Belgium) sets were used for all hybridization’s, in 4×44 K lay-out, covering 41174 Mus musculus 60-mer oligonucleotide probes, representing genes and transcripts. Hybridized slides were scanned on an Agilent scanner (G2565BA) at 100% laser power, 30% PMT. After data extraction using ImaGene 8.0 (BioDiscovery), print-tip Loess normalization was performed on mean spot intensities [25]. Data were analyzed using ANOVA [26] (R version 2.2.1/MAANOVA version 0.98–7; http://www.r-project.org/). P-values were determined by a permutation F2 test, in which residuals were shuffled 5000 times globally. Genes with P<0.05 after family-wise error correction (FWER) (or Benjamini-Hochberg correction/False discovery rate control (FDR)) were considered significantly changed. Details of the microarray data can be viewed at http://www.ncbi.nlm.nih.gov/geo (Gene Expression Omnibus accession number GSE45831).

## Results

### *Lmx1a* is Only Expressed during Development

Initial studies showed that *Lmx1a* expression starts around E8.5– E9 and remains expressed during life, although later studies suggested a decrease in *Lmx1a* expression already at P12 [27].

In order to elucidate the temporal window of *Lmx1a* activity, we performed in situ hybridization (ISH) experiments for *Lmx1a* and *Th* on adjacent mouse brain sections, collected directly after birth (P0), at P7 and at P14 (See Fig. S1). At P0, *Lmx1a* expression was found at high levels in the posterior hypothalamus (the P3 tegmentum), retromammillary area, subthalamic nucleus, substantia nigra pars compacta (SNc) and ventral tegmental area (VTA). At P7, *Lmx1a* transcript levels were clearly decreased in these regions, and at P14, *Lmx1a* expression was almost completely lost, which was in clear contrast to the retained high level of *Th* present in the mdDA region. When analyzing the expression in more detail in the different expression areas, the posterior hypothalamic area and retromammillary nucleus displayed a significantly lower expression level, although still present. In the VTA, expression was clearly lower and in the SNc only a few cells remained that express *Lmx1a* (Fig. S1, arrowheads). To conclude, the drastically lowering of *Lmx1a* transcript levels in mdDA neurons shortly after birth, suggests that *Lmx1a* is not involved in adult mdDA neuronal functions.

### *Lmx1a* is Required for Rostral mdDA Neurons

**Figure 1:**
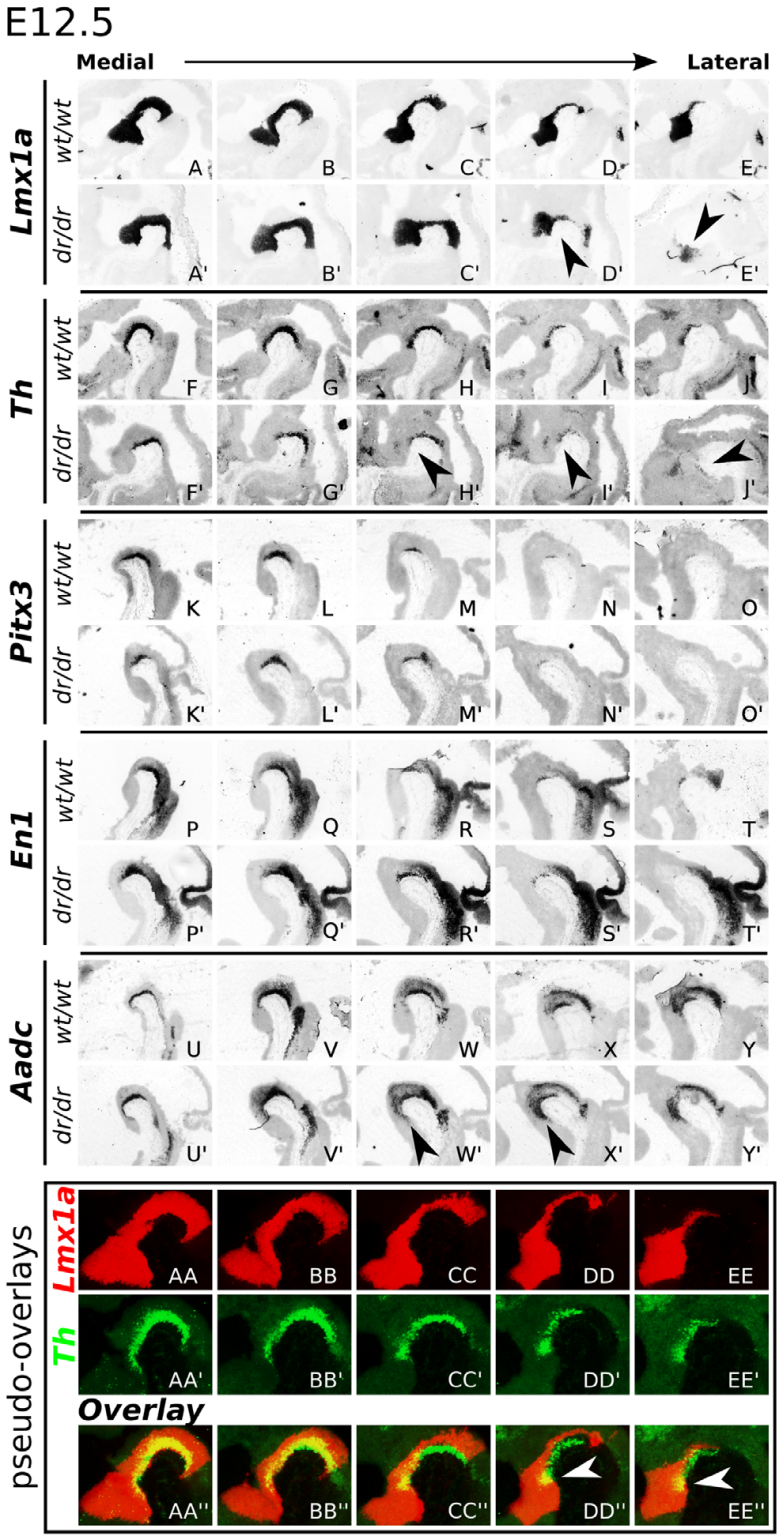
Phenotype characterization of E12.5 *Lmx1a-dr/dr* mice. (A–E′) Sagittal expression analysis of *Lmx1a* transcript in medial to lateral brain sections. Laterally, *Lmx1a* expression is clearly down-regulated in the *Dreher* homozygous mutant (D′,E′, arrowheads). (F–J′) *Th* transcript is rostral-laterally decreased (arrowheads), but also medially. (K–O′) *Pitx3* and (P–T′) *En1* display a subtle decreased expression in *Lmx1a-dr/dr* tissue. (U–Y′) *Aadc* expression is expanded rostrally in the absence of *Lmx1a* (arrowheads). (AA–EE′′) Pseudo-overlays of wild-type *Lmx1a* (red; generated from A–E) and *Th* (green; generated from F–J) transcript expression, showing complete overlap in medial sections and only partial overlap in lateral sections (white arrowheads).

To resolve the mdDA phenotype of the *Lmx1a* mutant, we characterized the *Lmx1a-dr/dr* mouse [18]in detail. Therefore, rostral-caudal and medial-lateral mapping by ISH was performed on E12.5 *Lmx1a-dr/dr* and wild-type sagittal sectioned tissue. Several mdDA markers, and *Lmx1a* itself were analyzed (Fig. 1). The defect in *Lmx1a* expression in the medial midbrain was only modest, marking the most caudal part. In contrast, in lateral sections of *Lmx1a-dr/dr*, a clear defect was observed in the diencephalon (P1, P2 and P3; [3], [28], where *Lmx1a* transcript levels were drastically lower (Fig. 1A–E′, arrowheads).

We found a subtle loss of *Th* expression in the medial midbrain (Fig. 1F–G′), which is in line with other studies [13], [16], [29], [30]. Interestingly, in rostral-lateral *Th* expression domains, a clear defect was observed. In this region *Th* transcript was almost absent, while in the control still a small set of *Th* positive cells was observed (Fig. 1F–J′, arrowheads). Also, for *Pitx3* and *En1* a small decrease in expression in the most lateral domains was displayed (Fig. 1K–O′ and P–T′), although this was more subtle in comparison to the loss of Th expression. Surprisingly, *Aadc* displayed clear expansion of its rostral expression domain (P3) (Fig. 1U–Y′, arrowheads). Since *Aadc* is suggested to be a marker for early differentiated mdDA neurons, this indicates that in the absence of *Lmx1a*, a rostral expansion of early, *Aadc* positive neurons might occur.

In order to visualize which mdDA region co-localizes with *Lmx1a* at E12.5, we created pseudo-overlays of *Lmx1a* and *Th* wild-type ISH images (Fig. 1AA–EE′). Medially, most *Th* expressing cells co-expressed *Lmx1a* (Fig. 1AA′′ and BB′′). However, more laterally *Lmx1a* was co-expressed with a small set of rostrally located *Th* expressing neurons only (Fig. 1DD′,EE′′, arrowheads). In contrast to the overlap of medial *Th* and *Lmx1a*, the loss of *Lmx1a* in this domain resulted in a very mild loss of *Th* expressing neurons. However, in the lateral domains, the rostral subset of *Th* neurons that co-expressed *Lmx1a*, apparently depend on this transcription factor, since this group was severely affected in the *Lmx1a-dr/dr mutant*.

### Requirement of *Lmx1a* in the Rostral mdDA Neuronal Field is Retained During Terminal Differentiation

**Figure 2:**
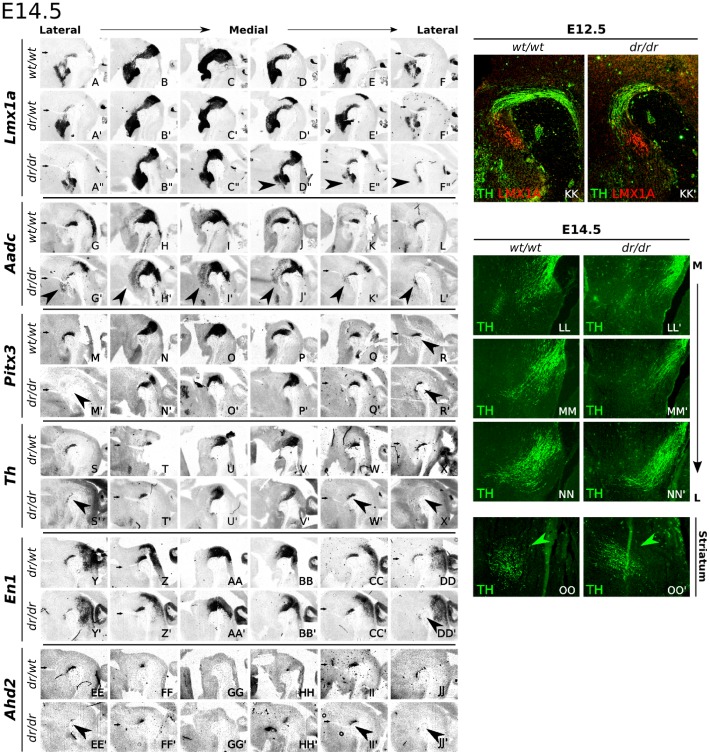
The observed rostral-lateral phenotype is retained in E14.5 *Lmx1a-dr/dr*. (AA–JJ′) ISH analysis of several mdDA markers in wild-type and *Lmx1a-dr/dr* tissue. Small arrows indicate the fasciculus retroflexus, as extra anatomical marker for comparing wild-type and mutant sections. (A–F′′) *Lmx1a* transcript levels are lowered in *Lmx1a-dr/dr* (large arrowheads), while between the heterozygous mutant and wild-type control no differences are observed. (G–L′) *Aadc* expression is laterally down-regulated, except for a clear rostral expansion of a group of cells in the diencephalon (G′K′,L′). Medially, *Aadc* expression is expanded rostrally and dorsally. (M–R′) *Pitx3* expression defects are found in the rostral-lateral parts were transcript levels are lower (arrowhead). (S–X′) A more pronounced defect is observed for *Th*, which is clearly reduced in lateral sections (arrowheads). (Y–DD′) *En1* expression is slightly decreased in most lateral domains. (EE–JJ′) For *Ahd2,* the most lateral expression domains are lost while more medial expression domains are unchanged. (KK–KK′) IHC analysis of E12.5 sagittal *Lmx1a* wild-type and knock-out tissue. The initial TH fiber outgrowth (green) appears unaffected in the mutant, since growth patterns towards LMX1A+ cells (red) are almost identical, despite a smaller number of TH+ cells. (LL–OO′) Medial to lateral analysis of TH fiber outgrowth and innervation within the striatum, in wild-type and *Lmx1a-dr/dr* tissue at E14.5, displays slightly fewer TH+ fibers in the knock-out. However, no clear phenotype is observed in direction or organization of the fibers, and they arrive at the expected site in the striatum (OO,OO′, green arrowheads).

In order to determine the significance of the initial E12.5 defect, we performed additional analysis on E14.5 *Lmx1a-dr/dr* and wild-type embryos, and it was revealed that the loss of rostral-lateral expression was retained in later developmental stages (Fig. 2A–DD′).

Since the affected *Th* expression seemed to be restricted to a rostral-lateral subset of mdDA neurons, we used *Ahd2* as a marker for this region [31]. Interestingly, and confirming the *Th* expression data, the affected rostral-lateral mdDA cell-group displayed a specific loss of *Ahd2*. At E14.5, the paramedian expression domain of *Ahd2* was unaffected (Fig. 2F–F′,H–H′), whilst the most rostral-lateral *Ahd2* domain was almost completely devoid of transcript (Fig. 2EE–JJ′, arrowheads). These observations suggest a subset-specific loss of *Th* and *Ahd2* in the rostral-lateral mdDA region. Importantly, not all of these affected cells are completely lost, since the mdDA markers *Pitx3* and *En1* are still present in a part of this rostral-lateral sub-population (Fig. 2P–R′ and Y–DD′).

Most prominent defects were consequently observed in the lateral-rostral part of the mdDA neuronal field. Therefore, possible defects in the *Th* fiber outgrowth were analyzed. TH immunohistochemistry was performed on E14.5 *Lmx1a-dr/dr* and wild-type tissue to follow fiber outgrowth in the diencephalic region (Fig. 2KK–OO′). The initial guidance direction of TH fibers seemed unchanged between *Lmx1a-dr/dr* and control (Fig. 2KK,KK′ and LL–NN′). To confirm normal striatal innervation, the arrival of TH bundles was analyzed at E14.5, and no obvious defects were observed (Fig. 2OO,OO′).

Taken together, *Lmx1a-dr/dr* shows medially a subtle defect, confirming previous reports [13], [16], [29]. Laterally, a rostral group of mdDA neurons (SNc) is clearly affected, as was observed for *Lmx1a*, *Th,* and *Ahd2*. Likely, the affected neurons are not completely lost at this stage, since *Pitx3* and *En1* were less affected, and *Aadc* expression was expanded, in this rostral group.

### LMX1A Expression is Restricted to the Rostral mdDA Region during Terminal Differentiation

The detailed ISH mapping of *Lmx1a-dr/dr* suggests a subset-specific requirement for *Lmx1a*, in the rostral-lateral domains of the mdDA neuronal field. To determine into more detail this rostral-lateral dependency, we analyzed the protein expression patterns of LMX1A and TH, in several mdDA developmental stages.

At E11.5, E12.0 and E12.5, medial expression of LMX1A protein was broad and fully overlapped the mdDA area (Fig. S2). Strikingly, when analyzing the expression pattern in the lateral TH domain, LMX1A protein was co-expressed in a rostral subset of TH neurons only (Fig. S2 D,G,H,I-J, arrowheads). In caudal-lateral mdDA neurons, a set of TH+ neurons did not express LMX1A (Fig. S2 I,J, asterisks), suggesting that there is a group of mdDA neurons that does not depend on LMX1A, during these developmental stages. Moreover, at E14.5 this restricted expression pattern was even more pronounced (Fig. S2 N–O). Medially, LMX1A was co-expressed in a small and select set of rostral TH+ neurons, whilst in the caudal mdDA area, no LMX1A expression was detected. Importantly, in lateral domains at this stage, TH expression largely co-localized with LMX1A protein (Fig. S2 P), suggesting a functional relationship in this area and developmental stage.

Taken together, during early mdDA differentiation, LMX1A is broadly present in TH+ cells. During terminal differentiation, LMX1A expression becomes restricted towards a subset of rostral (P3) and lateral TH+ neurons. Notably, this restricted co-localization underlines the observed phenotype in *Lmx1a-dr/dr*, where *Th*, and *Ahd2* are clearly affected in similar areas.

### Identification of Downstream Targets of *Lmx1a* via Loss-of-function Microarray Analysis *in vivo*

**Figure 3:**
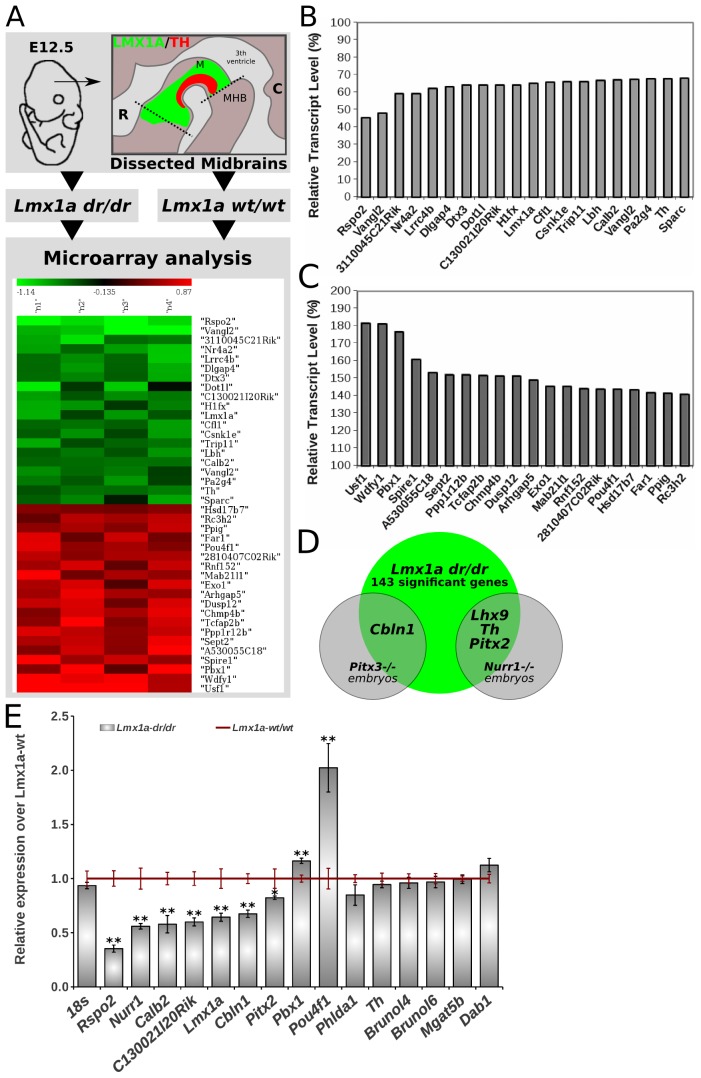
Genes regulated by *Lmx1a* in a microarray analysis of E12.5 *Lmx1a-dr/dr* embryos. (A) RNA was collected from E12.5 micro-dissected *Lmx1a-dr/dr* and *wt/wt* brains. *Lmx1a-dr/dr* samples were hybridized against *wt/wt* pooled RNA control. The heatmap represents up- (red) and down- (green) regulated genes based on log2-ratios of four individual microarray samples. Only the top 20 significantly up- and down-regulated genes are shown. (B) Relative expression levels of the 20 most up- and (C) down-regulated genes (microarray FWER ANOVA, p<0.05). With *Rspo2* as most down-regulated gene, and *Usf1* as highest up-regulated gene. (D) Venn-diagrams showing a number of genes that are also regulated in *Pitx3* and *NurrI* microarrays derived from previous studies. (E) qPCR validation of significant down-regulation of *Rspo2*, *Nurr1*, *Calb2*, *C130021l20Rik*, *Lmx1a*, *Cbln1* and *Pitx2*, and of significant up-regulation of *Pbx1* and *Pou4f1*, in *Lmx1a*-deficient embryonic midbrains. *18s* was used for normalization. Mean expression values in wt are set at 1 (red line) and are indicated with standard error bars (s.e.m.). Grey bars represent mean expression changes in *Lmx1a-dr/dr* samples compared to wt samples. Statistical analysis was performed with Student’s t-test. *P<0.05 is considered significant; **P<0.01. N = 4 for all analyzed genes and for each phenotype (each experimental sample (n) represents a pool of five micro-dissected midbrains). *M, midbrain; MHB, mid-hindbrain border; R, rostral; C, caudal; wt, wild-type.*

To elucidate the change in molecular programming as a result of the loss of *Lmx1a*, we performed microarray analysis on E12.5 mesodiencephalic and retromamillary tegmental (mdDA +RM) material of *Lmx1a-dr/dr*, compared to control littermates (Fig. 3A).

Microarray (ANOVA) analysis resulted in a total of 143 significantly regulated genes, of which 98 genes were down-regulated and 45 were up-regulated. Importantly, *Nurr1* (*Nr4a2*) and *Th* are among the 20 most down-regulated genes (Fig. 3A,B), confirming our phenotypic analysis. Interestingly, also the *Lmx1a* transcript level was 35% reduced. Furthermore, the most down-regulated gene was *Rspo2*, which transcript levels were reduced to 45% of wild-type levels. Among the 20 most up-regulated genes, *Usf1* was strongest up-regulated to 180% of wild-type levels (Fig. 3C). In addition, *Pbx1* was highly up-regulated, and interestingly, the red nucleus (RN) neuronal marker *Pou4f1* (*Brn3a*) was up-regulated as well, suggesting a suppressive effect of *Lmx1a* on this alternative RN fate. For subsequent analysis, a selection was made among all significantly regulated genes based on expression, and literature related to mdDA neurons (*Phlda1; Brunol4/6; Mgat5b*; *Nurr1; C130021l20Rik*; *Th; Calb2; Pbx1*), high fold-change (*Rspo2*) and migration (*Dab1*) (Table S3). In addition, *Pitx2* and *Cbln1* were selected based on their regulation by *Nurr1* and *Pitx3* respectively (Fig. 3D) [32], [33]. In order to validate our microarray data first, we subjected the RNA samples to qPCR analysis (Fig. 3E). Despite the previously observed subtle decrease of *Th* expression *in vivo* and in the microarray analysis, we could not confirm the down-regulation of this gene with the used qPCR method, and neither of *Phlda1*, *Brunol4*, *Brunol6*, *Mgat5b* and *Dab1*. Importantly, we confirmed clear down-regulation of *Rspo2*, *Nurr1*, *Calb2*, *C130021l20Rik*, *Lmx1a*, *Cbln1* and *Pitx2*. Furthermore, also the up-regulation of *Pbx1* and *Pou4f1* was confirmed.

### *Lmx1a* activates *Nurr1, Rspo2, Calb2* and *C130021L20RIK*

In order to identify the spatiotemporal regulation of the selected genes by *Lmx1a*, we performed ISH analysis in *Lmx1a-dr/dr* and wild-type mice at E12.5.

**Figure 4:**
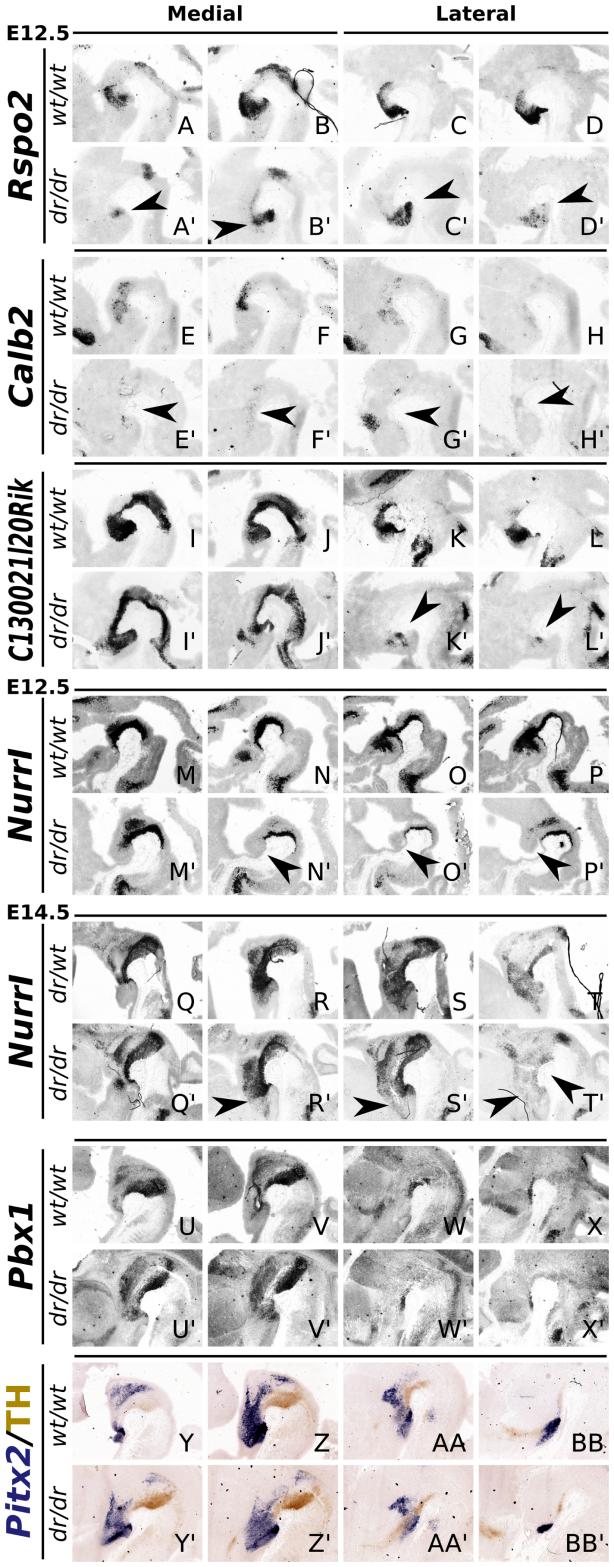
Validation of several *Lmx1a* target genes in *Lmx1a-dr/dr* embryos. (A–D′) ISH showing that *Rspo2* is drastically down-regulated in sagittal E12.5 *Lmx1a-dr/dr* tissue (arrowheads). (E–H′) *Calb2* expression is almost completely lost (arrowheads). (I–L′) *C130021L20Rik* is almost completely lost in lateral sections (arrowheads). (M–P′) Similarly, *Nurr1* expression is strongly down-regulated in rostral-lateral areas (arrowheads). (Q–T′) At E14.5, similar defects are observed; *NurrI* expression is clearly decreased in the rostral expression domain, specifically in lateral sections (arrowheads). (U–X′) *Pbx1* expression is slightly decreased in the most rostral-lateral domain. However, expansion of *Pbx1* expression is displayed in the rostral-medial area and dorsal to the medial mdDA region (in the red nucleus area). (Y–BB′) *Pitx2* expression (blue) level is down-regulated in rostral-medial areas. In the rostral-lateral domain, *Pitx2* expression is more clearly affected, as is displayed by a smaller *Pitx2* positive cell group. TH protein staining (brown) was taken along as a reference.

Small changes in the expression profile as a consequence of the loss of *Lmx1a,* were displayed for *Phlda1, Brunol4/6, Cbln1, Mgat5b* and *Dab1* (Fig. S3). *Mgat5b* displayed only subtle reduction of expression, in the lateral-caudal midbrain. For *Phlda1*, *Brunol4/6, Dab1* and *Cbln1*, expression was slightly diminished, mainly in rostral-lateral expression domains, and the latter two genes also displayed decreased expression in the lateral-caudal midbrain. Importantly, the other selected genes displayed a more striking loss of expression. At E12.5, the anterior midbrain/P1/P2/P3 plus RM area has lost *Nurr1* expression in *Lmx1a-dr/dr* mice (Fig. 4M–P′, arrowheads). A large part of this domain was outside the mdDA system, located in the RM hypothalamic area, however, also a rostral part of the mdDA neuronal field was affected. In addition, the remaining *Nurr1* expression in the mdDA area was reduced as well (Fig. 4N–O′). At E14.5, a similar phenotype was observed (Fig. 4Q–T′). Medially, the overall levels of *Nurr1* were slightly affected in *Lmx1a-dr/dr*. Laterally, the anterior segment was severely affected and in the most lateral mdDA domain, *Nurr1* expression was almost absent (Fig. 4S–T′; Fig. S4). Interestingly, comparable deficits were observed for *Pitx2* (Fig. 4Y–BB′), *C130021l20Rik* (Fig. 4I–L′), *Calb2* (Fig. 4E–H′) and *Rspo2* (Fig. 4A–D′). *C130021L20Rik* showed a decrease in the anterior-medial brain area. Furthermore, the transcript was almost completely abolished in the RM hypothalamic area (Fig. 4K–L′′). C*alb2* expression was widely affected in the *Lmx1a-dr/dr*. Medially, levels were significantly decreased (Fig. 4E–F′), and laterally almost all *Calb2* expression was lost (Fig. 4G–H′). For *Rspo2,* a marked decrease in expression was observed. In medial brain areas, the number of *Rspo2* expressing cells was significantly lower, mainly in the rostral part (P3) (Fig. 4A–B′). In addition, in the lateral domains, the anterior domain was clearly reduced (Fig. 4C–D′), and in the most lateral *Rspo2* expression domains, completely lost (Fig. S5).

Taken together, the genes discussed above are all influenced by *Lmx1a* activity *in vivo* and the most marked deficits were observed for *Nurr1, C130021L20Rik, Calb2* and *Rspo2*. These findings strongly suggest that these genes are direct or indirect down-stream targets of *Lmx1a*. Moreover, the *Lmx1a* dependency is region-specific and seems most severe for the rostral-lateral expression domains (P3 and the RM hypothalamic area).

### *Calb2* Expression is Selectively Affected in the Adult Medial-rostral SNc and Medial-dorsal VTA

**Figure 5:**
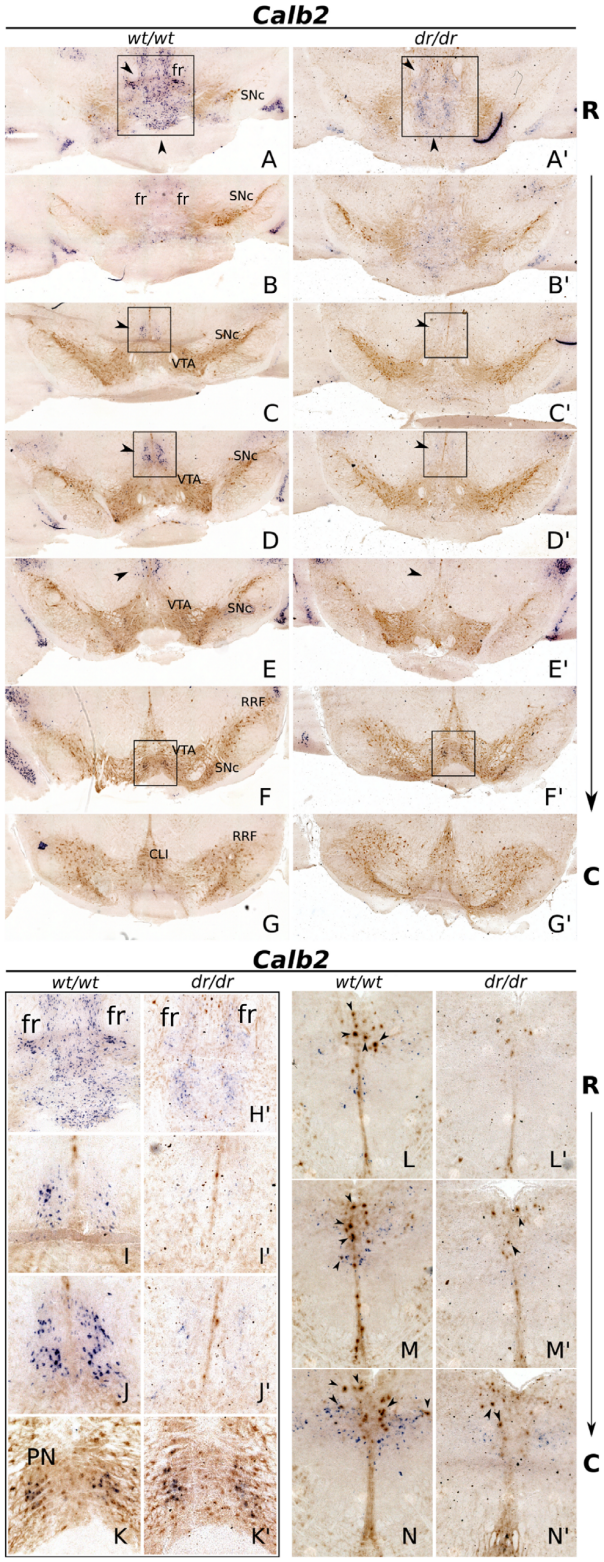
*Calb2* expression is selectively affected in adult *Lmx1a-dr/dr*. (A–G′) *Calb2* mRNA expression (blue) combined with TH protein IHC staining (brown). Rostrally, *Calb2* expression is drastically lower in the medial midbrain (A,A′, arrowheads) and (H–H′). In the medial VTA a dorsal *Calb2* positive group is observed in the wild-type, whilst in the knock-out, this expression is almost completely lost (C–E′, arrowheads) and (I–J′). In other regions, *Calb2* expression appears unaffected. In the medial and caudal VTA, more ventrally, two small *Calb2* expressing cell groups are identified in wild-type, that appear unchanged in knock-out tissue (F,F′) and (K–K′). (L–N′) *Calb2* expression ventrally of the aqueduct, in the periaqueductal gray, is clearly decreased in the absence of *Lmx1a*. A subset of TH expressing neurons co-expresses *Calb2* and most of these cells are lost in *Lmx1a-dr/dr* (arrowheads). *SNc, Substantia nigra pars compacta; VTA, ventral tegmental area; RRF, retrorubral field; CLI, central linear nucleus; fr, fasciculus retroflexus; mtg, mammillotegmental tract; PN, nucleus paranigralis; R, rostral; C, caudal.*

During development, *Calb2* was severely affected in the absence of *Lmx1a*, not only in the rostral-lateral areas but also in the medial midbrain domain. To asses whether this loss is maintained in the mature mdDA system, we performed ISH analysis on adult coronal *Lmx1a-dr/dr* and wild-type material (Fig. 5).

In the mature midbrain, *Calb2* was highly expressed medially in the most rostral domain of the diencephalon, located mostly between the rostral SNc groups, with a slight enrichment of *Calb2* expression around the fasciculus retroflexus (fr) and the mammillotegmental tract (mtg). Interestingly, in *Lmx1a-dr/dr* sections, *Calb2* expression was drastically reduced in this area (Fig. 5A,A′,H,H′). Furthermore, in the medial dorsal VTA, a *Calb2* positive group clearly being expressed in the wild-type brain, was almost completely lost in the absence of *Lmx1a* (Fig. 5C–E′,I–J′). The loss of *Calb2* expression seemed selective since in the more caudal-ventral VTA, close to the nucleus paranigralis (PN), two *Calb2* expressing cell-groups were clearly observed in both wild-type and *Lmx1a-dr/dr* tissue, with no visible change in expression levels (Fig. 5F,F′,K,K′). Additionally, more dorsal of the medial mdDA system, located directly ventral of the aqueduct, in the periaqueductal gray, *Calb2* positive cells were observed in wild-type tissue. In this same area, a number of TH positive neurons was present as well, and part of these neurons co-expressed *Calb2* (Fig. 5L,M,N, arrowheads). Interestingly, in the absence of *Lmx1a*, a drastic decrease in *Calb2* expression was shown, together with a clear decrease in TH positive neurons; in *Lmx1a-dr/dr* tissue, a clear decrease in *Calb2*/TH co-expressing neurons was observed (Fig. 5L′,M′,N′, arrowheads).

In conclusion, the domain-specific reduction of *Calb2* at selective sites in the adult midbrain, hint towards a region-specific dependency on *Lmx1a*, during development.

### Early Failure of *Rspo2*, *NurrI* and *C130021L20Rik* Marks the Loss of Rostral-lateral Positioned Adult mdDA Neurons

*Th, Nurr1*, *Rspo2*, and *C130021L20Rik* were all drastically down-regulated in *Lmx1a-dr/dr* tissue during development, mainly in the rostral-lateral mdDA neuronal region and in retromammilary areas of the hypothalamus. In order to asses the consequences of this early expression aberration, we analyzed the mdDA neuronal field in more detail in coronal sections of *Lmx1a-dr/dr* and wild-type adult brains.

**Figure 6:**
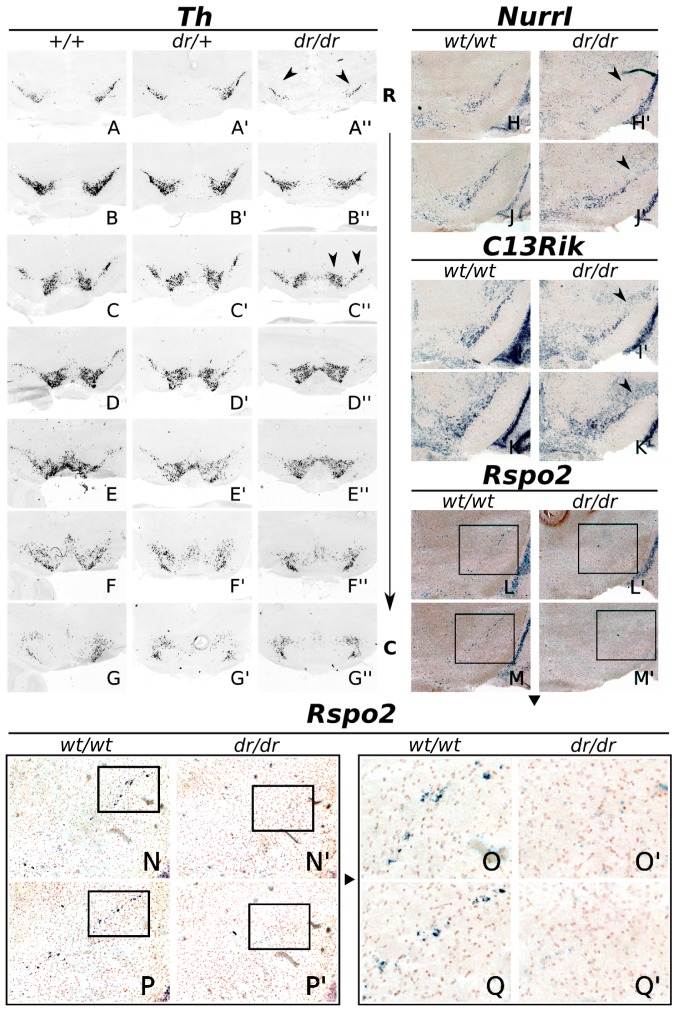
Expression of *Th, NurrI*, *C130021L20Rik* and *Rspo2* is affected in the adult SNc. (A–G′′) *Th* expression in the adult midbrain from rostral to caudal, marking the SNc and the VTA respectively. A comparison between wild-type (wt), heterozygous and homozygous *Dreher* material demonstrates almost identical expression patterns between *wt/wt* and *wt/dr* coronal brain sections, whilst obvious defects can be seen in the rostral-lateral part of the SNc in *dr/dr* midbrains (arrowheads). The medial VTA displays some reduction, although more subtle than in rostral-lateral mdDA domains. (H–K′) Expression of *NurrI* (H,J,H′,J′), and *C130021L20Rik* (I,K,I′,K′), shows similar defects as was seen for *Th*; rostral-lateral expression is diminished (arrowheads). (L–Q′) A complete loss of *Rspo2* expression in the SNc was observed. In the wild-type, only a subset of cells in the SNc domain express *Rspo2*, and all *Rspo2* expression is lost in the affected SNc of the *Lmx1a* knock-out, as can be observed in higher magnifications (O,Q,O′,Q′).

In line with the observed *Th* defects in E12.5 and E14.5 tissue, in the adult *Lmx1a-dr/dr* a clear decrease in *Th* expression was observed (Fig. 6A–G′′). The rostral-lateral domain of the SNc showed a complete loss of *Th* expression. Moreover, the remaining part of SNc was clearly smaller. Furthermore, a subtle reduction in *Th* expression in the medial VTA region was observed which confirmed earlier published data on this mutant [17]. In agreement with the decrease in *Th* expression in adult *Lmx1a-dr/dr* material, *Nurr1*, *C130021L20Rik* and *Rspo2* were reduced in the rostral lateral SNc (Fig. 6H–Q′). Importantly, in the absence of *Lmx1a*, *Rspo2* expression was completely lost in the adult mdDA system. In wild-type, a small but specific set of *Rspo2* positive cells was present throughout the SNc, and some cells in the region between VTA and hypothalamus (Fig. 6L–M′) and more caudally, in the lateral VTA (data not shown). In *Lmx1a-dr/dr* tissue, *Rspo2* positive cells were lost in the SNc, VTA and hypothalamic area (Fig. 6L′–Q′; and data not shown).

In order to confirm that *Rspo2* expression is specifically localized within mdDA neurons, we performed combined ISH/IHC for *Rspo2* transcript and TH protein (Fig. S6). Intriguingly, most cells expressing *Rspo2* were observed in the SNc. Importantly, all *Rspo2* positive cells in this area co-expressed TH protein (Fig. S6, arrowheads). In addition to some hypothalamic expression in non-DA neurons (data not shown), *Rspo2* was in the midbrain uniquely expressed in a subset of mdDA neurons in the VTA and SNc. Interestingly, Rspo2 expression was enriched in the rostral-lateral domains of the SNc (Fig. S6 A′′–D′′, arrowheads), areas that are clearly affected in *Lmx1a* mutants.

To conclude, *Rspo2* is expressed in a subset of mdDA neurons, which is lost in *Lmx1a-dr/dr* mutants. The loss of *Rspo2* expression in a small number of VTA neurons, and a larger number of SNc neurons, underlines the *Th* phenotype in these areas, suggesting that in the adult stage these specific mdDA neurons not only lose Th expression, but are lost completely as a consequence of mdDA identity loss.

### A Subset of Developing mdDA Neurons is Affected in Embryonic *Rspo2* knock-out Mice

To examine if the mdDA phenotype observed in *Lmx1a*-dr embryos, might be caused by the loss of *Rspo2*, we analyzed several mdDA markers in E14.5 sagittal midbrain sections of *Rspo2-LacZ* knock-out mice [20], compared to littermate controls.

**Figure 7:**
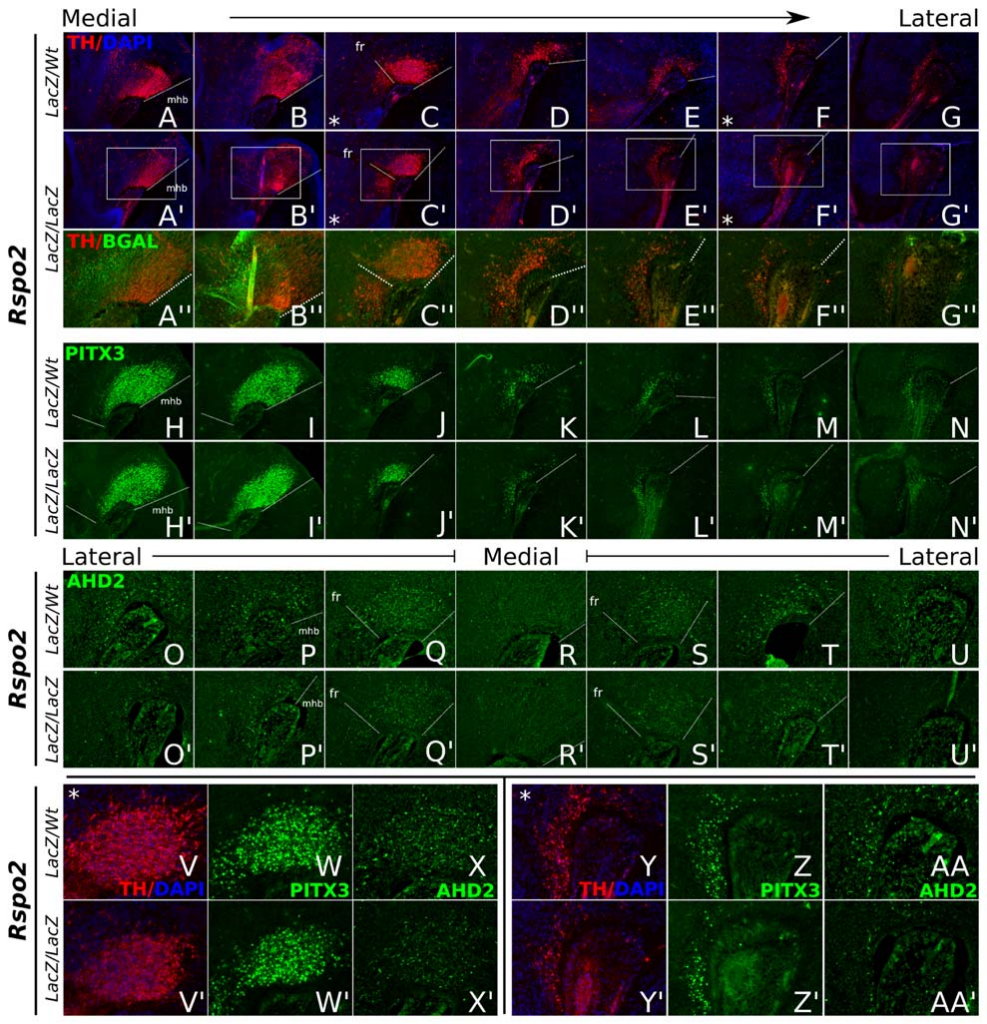
mdDA markers TH, AHD2 and PITX3 display loss of expression in *Rspo2-LacZ/LacZ* embryonic midbrain. Sagittal sections of *Rspo2* control and knock-out (*LacZ/LacZ*) littermate mouse brains, at E14.5. (A–G′) TH protein expression analysis reveals a decrease in TH+ cells in the *Rspo2*-ablated mdDA neuronal field. Lines indicating the mid-hindbrain border (mhb) and the fasciculus retroflexus (fr) are added for clarity. (A′′–G′′) More detailed images of (A′–G′). BGAL protein staining can be observed in *Rspo2-LacZ/LacZ* mutant cells. Most of these cells do not express TH (anymore). (H–N′) A decrease in PITX3-expressing mdDA neurons in *Rspo2-LacZ/LacZ* tissue is shown. (O–U′) From lateral to medial to lateral, AHD2 analysis reveals a decrease in expression in the paramedian and lateral mdDA neuronal subset, in the absence of *Rspo2*. (V–X′) Higher magnifications of (C,C′), (J,J′) and (Q,Q′), showing a mild but clear decrease of mdDA neurons expressing TH, PITX3 and AHD2 in the paramedian midbrain. (Y–AA′) Higher magnifications of (F,F′), (M,M′) and (O,O′), showing a clear decrease in the number of TH-, PITX3-, and AHD2-positive neurons in the lateral mdDA neuronal field.

TH protein expression analysis revealed a small decrease (65% of wt, data not shown) in TH+ cells in the rostral/lateral mdDA neuronal field (Fig. 7A–G′,V–V′,Y–Y′). Furthermore, in these affected areas, BGAL expression was observed, suggesting that the loss of TH expression overlaps with the position of otherwise *Rspo2* positive neurons (Fig. 7A′′–G′′). Similarly, a small but clear decrease of PITX3+ neurons was observed, suggesting that the affected neurons lose their mdDA identity (Fig. 7H–N′,W–W′,Z–Z′). In line with this, AHD2 expression analysis revealed a comparably decreased expression domain (Fig. 7O–U′,X–X′,AA–AA′). This might indicate that two situations occur in the absence of Rspo2: possible loss of neurons as presented by the subtle loss of TH and PITX3 expressing cells, and in addition loss of mdDA coding as shown by affected AHD2 expression in these areas. Remaining BGAL staining and normal Dapi-staining suggest that no massive cell loss occurred in the affected region.

The identification of *Nurr1* and *Rspo2* as down-stream targets of *Lmx1a*, made it tempting to hypothesize that one of these factors is maybe regulated by the other. We therefore investigated *Rspo2* and *Nurr1* expression in E14.5 *Nurr1* and *Rspo2* knock-out brains, respectively. *Rspo2* was normally expressed in the absence of *Nurr1* (data not shown). Also, in *Rspo2-LacZ/LacZ* tissue, *Nurr1* expression was hardly affected (data not shown). This indicated that *Rspo2* and *Nurr1* are not regulated by each other.

## Discussion

### Novel Targets of *Lmx1a* Implicated in Neuronal Differentiation and Identity

Many studies have suggested an essential role for *Lmx1a* in mdDA neuronal development. This was shown by loss-and-gain-of-function studies, in chick embryos [5], and by analyzing markers in the *Lmx1a* mutant (*Lmx1a-dr/dr)* [13], [16]. In addition, the essential role of *Lmx1a* in dopaminergic differentiation of stem cells was shown [5], [15], [34]–[37]. In literature, it is suggested that *Lmx1a* plays a role in proliferation and neurogenesis, but despite the indication that several factors are regulated by *Lmx1a*, like *NurrI*, *Msx1* and *Wnt1*, the precise role of *Lmx1a* in the mouse is still not clearly identified. In order to unravel the exact molecular programming activated by *Lmx1a*, we performed an *in vivo* transcriptome analysis by using *Lmx1a-dr/dr* embryonic mdDA plus RM brain areas.

**Figure 8:**
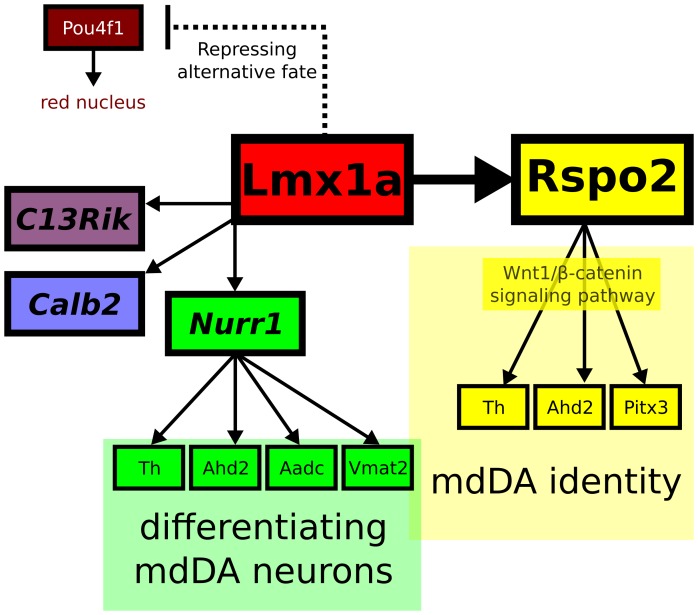
A model integrating several identified targets of *Lmx1a*. *Pou4f1* (*Brn3a*) was strongly up-regulated in the *Lmx1a−/−* expression array, in line with a suggested role of *Lmx1a* in repressing an alternative, red nucleus, fate during development (dashed line). *Lmx1a* targets *C130021l20Rik* and *Calb2* require *Lmx1a* directly or indirectly for correct expression in subsets of the mdDA system. The *Nurr1* transcriptional pathway induces differentiating mdDA neurons, and loss of *Lmx1a* resulted in affected *Nurr1* expression, but also in affected expression of *Nurr1* transcriptional targets, mainly in rostral-lateral mdDA neurons. *Rspo2* is involved in the *Wnt1*/b-catenin signaling pathways, which is implicated in proliferation and cell-cycle exit. Loss of *Lmx1a*, resulted in decreased *Rspo2* expression and loss of mdDA neuronal markers as Th, Ahd2 and Pitx3. Interestingly, loss of *Rspo2* partially phenocopied the defects observed in the *Lmx1a-dr/dr* mdDA system.

During development, one of the domains that arises adjacent to the mdDA neuronal field, is the red nucleus (RN). And in several studies, it was observed that *Lmx1a* can repress *Pou4f1*, and in the absence of *Lmx1a*, an up-regulation of *Pou4f1* was observed [16], [29]. Confirming this, in our microarray analysis *Pou4f1* was found in the top of significantly up-regulated genes, and displayed more than two-fold up-regulation with qPCR (Fig. 3). This regulation might mark or even influence the neuronal programming towards a RN phenotype instead of a DA phenotype (Fig. 8). Furthermore, we observed an expansion of *Pbx1* towards the mdDA neighboring neuronal fields (Fig. 4), further suggesting changes in neuronal identities in these developing domains due to lack of repression by *Lmx1a*.

Importantly, we revealed that the *Lmx1a*-regulated genes *Nurr1*, *Calb2*, *C130021L20Rik* and *Rspo2* are drastically down-regulated outside and in the mdDA area, in *Lmx1a-dr/dr* embryos (Fig. 8). Moreover, a clear deficit of *Th, Nurr1*, *C130021L20Rik* and *Rspo2* expression was identified in the adult rostral-lateral SNc, whereas *Calb2* expression was affected in medial domains, mostly adjacent to the mdDA system, with only minor defects in TH expression in this area. Interestingly, *Calb2* (Calretinin) is a calcium ion binding protein expressed in GABA-ergic neurons and it can be considered as a marker for early neuronal differentiation [38], [39]. The observed loss of *Calb2* expression in *Lmx1a-dr/dr*, suggests that these cells have a changed calcium homeostasis and therefore lack the proper programming which should normally be present in the *Calb2* expressing subset of mdDA neurons. Despite a drastic decrease in *Calb2* expression in the *Lmx1a-dr/dr* embryonic brain, the consequences for the fully developed mdDA system seem only modest, since few TH positive cells are lost, in small and selective domains of the medial mdDA area only.

The *Lmx1a*-regulated gene *C130021L20Rik* is a large intervening non-coding RNA molecule (lincRNA). It was discovered recently that many of these lincRNA’s are transcriptionally regulated and moreover, are involved in regulation of gene transcription [40]. Interestingly, the open reading frame of *C130021L20Rik* is located adjacent to the open reading frame of *Lmx1b*, and the developmental and adult expression pattern is very similar to the expression pattern of *Lmx1b* (and *Lmx1a*).

In the absence of *Lmx1a*, *Nurr1* expression is drastically down-regulated, in the anterior midbrain/P1/P2/P3 plus RM domains. Together with the high fold-change down-regulation of its transcript levels in the microarray study and qPCR analysis, this strongly indicates that this key mdDA factor is a downstream target of *Lmx1a*. Therefore, the subtle defects found for *Th* in the developing mdDA system in the absence of *Lmx1a*, are probably a consequence of affected *Nurr1* expression (Fig. 8).

### *Lmx1a* Regulates *Wnt* Modulator *Rspo2* in a Subset of mdDA Neurons

Like *Nurr1,* the gene *Rspo2* was severely affected due to the absence of *Lmx1a*, during development and in adult mdDA neurons. *Rspo2* belongs to the group of R-spondins, a family of secreted proteins that activate *Wnt*/b-catenin signaling [41]–[43]. In mice, from E8.5 all four known Rspo’s are expressed, displaying a complex expression pattern throughout the whole embryonic body [20]. *Rspo2*-null mice die immediately after birth because of respiratory failure, and embryos display defects in the limbs (distal limb loss), craniofacial structures (cleft palate and mild facial skeletal defects), lung hypoplasia and pulmonary vascular defects [44]–[46]. Interestingly, it was shown that all four R-spondin members regulate *Wnt* signaling at the level of the *Frizzled8* and *Lrp6* receptors [47], [48]. Another study reported that *Rspo2* modulates *Wnt* signaling in mouse mammary epithelial cells, and that *Rspo2* and *Wnt1* act synergistically in the b-catenin pathway [49]. Rspo’s can stabilize the level of cytosolic b-catenin and synergize with *Wnt-*ligands in order to promote b-catenin transcriptional activity [47], [50], [51]. Intriguingly, *Wnt1* is important for proliferation and patterning of the mdDA neuronal field [52], and several papers suggested a role of *Lmx1a* together with *Wnt1* in mdDA differentiation. Recently, a novel *Wnt1*-*Lmx1a* auto-regulatory loop was identified during mdDA differentiation of mouse ES cells [15]. Moreover, it was described that the reduced number of mdDA progenitors in *Lmx1a-dr/dr* and *Lmx1a*/*Lmx1b* double mutants may be a consequence of proliferation defects and an increase in cell cycle exit of the progenitors [16]. Also, their data indicate that *Wnt1* expression was slightly reduced in *Lmx1a-dr/dr*, and specifically lost in the *Lmx1a*/*Lmx1b* double mutant, suggesting that both genes specifically and redundantly regulate *Wnt1* expression in the mdDA domain [16]. Since *Rspo2* acts in the *Wnt1*/b-catenin signaling pathway, a reduction in *Rspo2* expression might influences the end result of *Wnt* activity. We speculate that the lack of *Rspo2* protein in the affected neurons of the *Lmx1a-dr/dr* mutant might induce the previously suggested early cell-cycle exit and premature differentiation. In line with this, in the *Lmx1a-dr/dr* mutant we observed in the current study a clear expansion of *Aadc* expression, which might represent an expansion of early mdDA neurons. However, it may also display an induction of other monoaminergic neuronal phenotypes in this area.

Additionally, when comparing the loss of mdDA neurons (or a loss of mdDA identity) between the *Lmx1a* and the *Rspo2* mutant, we found that the observed defects are partially identical between both mutants, where the terminal differentiation markers Th and Pitx3 showed the same subtle reduced expression. It is therefore likely that part of the *Lmx1a*-phenotype is caused by the loss of down-stream target *Rspo2*, affecting a number of mdDA neurons positioned in the VTA and more prominently in the SNc.

### Conclusions

We have shown that *Lmx1a* is essential for a rostral-lateral subset of developing mdDA neurons, and loss of *Lmx1a* results in loss of the correct neuronal identity, leading to an affected SNc in the adult mouse brain. Transcriptome analysis of *Lmx1a*-deficient embryonic brains revealed several genes that are known for their role in mdDA function and development, such as *Th* and *Nurr1*. In addition, several novel genes were identified, leading to a better understanding of the subset-specific phenotype of the *Lmx1a*-deficient mdDA system, since these genes are mainly depending on *Lmx1a* in the rostral-lateral mdDA neuronal field. Moreover, loss of the highly regulated *Lmx1a*-dependent gene *Rspo2*, partially phenocopied the *Lmx1a*-mutant. To conclude, in dept characterization and gene expression profiling of *Lmx1a*-deficient mdDA neurons, provided a detailed map of the molecular pathway in which *Lmx1a* is acting towards the correct development of mdDA neuronal subsets.

## Supporting Information

Figure S1 ***Lmx1a* expression is down-regulated shortly after birth.** Coronal sections of wild-type mouse tissue at P0, P7 and P14. In situ hybridization of *Lmx1a* and *Th* is shown from rostral to caudal. *Th* was taken along to mark the SNc and VTA and as a control for transcript levels. (A–F) A significant loss of *Lmx1a* expression is observed in the SNc at P14, when compared to P7 or P0, and when compared to *Th*. (A′,C′,E′) *Lmx1a* expression in the SNc in more detail, showing that only few cells remain that express *Lmx1a*, in low levels. (G–R) Both in caudal SNc, and more caudally, the VTA, the expression of *Lmx1a* is clearly diminished at P14 (arrowheads). *P, postnatal day; SNc, Substantia nigra pars compacta; VTA, ventral tegmental area*.
(TIF)

Figure S2 **LMX1A protein expression is restricted to rostral-lateral TH expression at later developmental stages.** (A–H) IHC on E11.5 (A–D) and E12.0 (E–H) sagittal wild-type mouse tissue, showing LMX1A protein (red) and TH protein (green). Medially, full protein overlap is displayed, whereas in lateral expression domains, only a subset of TH+ neurons overlaps with LMX1A. (I–J) Higher magnifications of G and H, showing the group of TH+ neurons that co-localize with LMX1A (white arrowheads), and a group of TH expressing neurons that do not express LMX1A (green asterisks). (K–M) The observed rostral-lateral overlap is more clear at E12.5, where also in the medial brain, a rostral specificity of LMX1A occurs. (N–P) At E14.5, the rostral LMX1A/TH restriction is clearly observed in medial and lateral midbrain areas.
(TIF)

Figure S3 ***Lmx1a* regulates P** ***hlda1, Brunol4, Brunol6, Cbln1, Mgat5b* and *Dab1* in selective areas of the *Lmx1a*-expression domain.** Sagittal *Lmx1a* control and *Lmx1a-dr/dr* sections, medial and lateral. On the right, higher magnifications of the boxed areas are shown. (A–E′) *Phlda1* expression is slightly reduced in rostral parts of the brain (arrowheads). (F–O′) *Brunol4* and *Brunol6* both show a slight down-regulation in rostral-lateral domains (G,G′, and arrowheads). (P–T′) *Cbln1* expression is diminished mainly in rostral areas. (U–Y′) *Mgat5b* transcript levels are slightly reduced in the lateral-caudal midbrain (arrowheads). (Z–DD′) *Dab1* shows loss of expression in medial-rostral areas (Z′,AA′,DD′, arrowheads). Also lateral-caudal, expression is affected (CC′, arrowhead).
(TIF)

Figure S4 ***Lmx1a* and *NurrI* expression throughout the sagittal brain at E14.5.** *Lmx1a* expression (left columns) from lateral (L) to medial (M) to lateral brain domains, in wild-type and *Lmx1a-dr/dr* tissue, at E14.5. In lateral domains, *Lmx1a* expression is down-regulated in the *Lmx1a* knock-out, and in all sections throughout the brain, a rostral defect is shown. *NurrI* expression (right columns) was analyzed in the same set-up. For *NurrI* a drastically decrease in rostral and lateral expression can be observed (arrowheads).
(TIF)

Figure S5 ***Lmx1a* and *Rspo2* expression throughout the sagittal brain at E12.5.** *Lmx1a* expression (left columns) from lateral (L) to medial (M) to lateral brain domains, in wild-type and *Lmx1a-dr/dr* tissue, at E12.5. In lateral positions, *Lmx1a* expression is clearly down-regulated in the *Lmx1a* knock-out, and in all sections, a rostral defect can be seen. *Rspo2* expression (right columns) was analyzed in the same set-up. For *Rspo2* an even more drastic decrease in rostral and lateral expression can be observed (arrowheads).
(TIF)

Figure S6 ***Rspo2* co-localizes with TH in adult mdDA neurons.** (A–C) Combined ISH/IHC for *Rspo2* (blue) and TH (brown). Most *Rspo2-*positive cells are found in the rostral and lateral mdDA system, in the SNc (A′′,B′′,C′′, arrowheads) and also some cells expressing *Rspo2* are observed in the VTA (A′,B′,C′). All *Rspo2-*positive cells also express TH, indicating that *Rspo2* is expressed in a subset of mdDA neurons (D).
(TIF)

Table S1 **List of primers used for the generation of in situ hybridization probes.**
(XLS)

Table S2 **List of primers used for qPCR.**
(XLS)

Table S3 **A selection of genes regulated in E12.5 *Lmx1a-dr/dr* mouse embryos.**
(PDF)

## References

[1] Petros TJ, Tyson JA, Anderson SA (2011) Pluripotent stem cells for the study of CNS development. Front Mol Neurosci 4: 30 doi:10.3389/fnmol.2011.00030 22016722 10.3389/fnmol.2011.00030 PMC3191505

[2] Prakash N, Wurst W (2006) Genetic networks controlling the development of midbrain dopaminergic neurons. J Physiol (Lond) 575: 403–410 doi:10.1113/jphysiol.2006.113464 16825303 10.1113/jphysiol.2006.113464 PMC1819467

[3] Smits SM, Burbach JPH, Smidt MP (2006) Developmental origin and fate of meso-diencephalic dopamine neurons. Prog Neurobiol 78: 1–16 doi:10.1016/j.pneurobio.2005.12.003 16414173 10.1016/j.pneurobio.2005.12.003

[4] Andersson E, Jensen JB, Parmar M, Guillemot F, Björklund A (2006) Development of the mesencephalic dopaminergic neuron system is compromised in the absence of neurogenin 2. Development 133: 507–516 doi:10.1242/dev.02224 16396906 10.1242/dev.02224

[5] Andersson E, Tryggvason U, Deng Q, Friling S, Alekseenko Z, et al (2006) Identification of intrinsic determinants of midbrain dopamine neurons. Cell 124: 393–405 doi:10.1016/j.cell.2005.10.037 16439212 10.1016/j.cell.2005.10.037

[6] Ferri ALM, Lin W, Mavromatakis YE, Wang JC, Sasaki H, et al (2007) Foxa1 and Foxa2 regulate multiple phases of midbrain dopaminergic neuron development in a dosage-dependent manner. Development 134: 2761–2769 doi:10.1242/dev.000141 17596284 10.1242/dev.000141

[7] Saucedo-Cardenas O, Quintana-Hau JD, Le WD, Smidt MP, Cox JJ, et al (1998) Nurr1 is essential for the induction of the dopaminergic phenotype and the survival of ventral mesencephalic late dopaminergic precursor neurons. Proc Natl Acad Sci USA 95: 4013–4018. 9520484 10.1073/pnas.95.7.4013 PMC19954

[8] Smidt MP, Van Schaick HS, Lanctôt C, Tremblay JJ, Cox JJ, et al (1997) A homeodomain gene Ptx3 has highly restricted brain expression in mesencephalic dopaminergic neurons. Proc Natl Acad Sci USA 94: 13305–13310. 9371841 10.1073/pnas.94.24.13305 PMC24304

[9] Smidt MP, Asbreuk CH, Cox JJ, Chen H, Johnson RL, et al (2000) A second independent pathway for development of mesencephalic dopaminergic neurons requires Lmx1b. Nat Neurosci 3: 337–341 doi:10.1038/73902 10725922 10.1038/73902

[10] Smidt MP, Smits SM, Burbach JPH (2004) Homeobox gene Pitx3 and its role in the development of dopamine neurons of the substantia nigra. Cell Tissue Res 318: 35–43 doi:10.1007/s00441-004-0943-1 15300495 10.1007/s00441-004-0943-1

[11] Kele J, Simplicio N, Ferri ALM, Mira H, Guillemot F, et al (2006) Neurogenin 2 is required for the development of ventral midbrain dopaminergic neurons. Development 133: 495–505 doi:10.1242/dev.02223 16410412 10.1242/dev.02223

[12] Smits SM, Smidt MP (2006) The role of Pitx3 in survival of midbrain dopaminergic neurons. J Neural Transm Suppl: 57–60. 10.1007/978-3-211-45295-0_10 17017509

[13] Ono Y, Nakatani T, Sakamoto Y, Mizuhara E, Minaki Y, et al (2007) Differences in neurogenic potential in floor plate cells along an anteroposterior location: midbrain dopaminergic neurons originate from mesencephalic floor plate cells. Development 134: 3213–3225 doi:10.1242/dev.02879 17670789 10.1242/dev.02879

[14] Puelles E, Annino A, Tuorto F, Usiello A, Acampora D, et al (2004) Otx2 regulates the extent, identity and fate of neuronal progenitor domains in the ventral midbrain. Development 131: 2037–2048 doi:10.1242/dev.01107 15105370 10.1242/dev.01107

[15] Chung S, Leung A, Han B-S, Chang M-Y, Moon J-I, et al (2009) Wnt1-lmx1a forms a novel autoregulatory loop and controls midbrain dopaminergic differentiation synergistically with the SHH-FoxA2 pathway. Cell Stem Cell 5: 646–658 doi:10.1016/j.stem.2009.09.015 19951692 10.1016/j.stem.2009.09.015 PMC2788512

[16] Yan CH, Levesque M, Claxton S, Johnson RL, Ang S-L (2011) Lmx1a and lmx1b function cooperatively to regulate proliferation, specification, and differentiation of midbrain dopaminergic progenitors. J Neurosci 31: 12413–12425 doi:10.1523/JNEUROSCI.1077-11.2011 21880902 10.1523/JNEUROSCI.1077-11.2011 PMC6703256

[17] Deng Q, Andersson E, Hedlund E, Alekseenko Z, Coppola E, et al (2011) Specific and integrated roles of Lmx1a, Lmx1b and Phox2a in ventral midbrain development. Development 138: 3399–3408 doi:10.1242/dev.065482 21752929 10.1242/dev.065482

[18] Millonig JH, Millen KJ, Hatten ME (2000) The mouse Dreher gene Lmx1a controls formation of the roof plate in the vertebrate CNS. Nature 403: 764–769 doi:10.1038/35001573 10693804 10.1038/35001573

[19] Chizhikov V, Steshina E, Roberts R, Ilkin Y, Washburn L, et al (2006) Molecular definition of an allelic series of mutations disrupting the mouse Lmx1a (dreher) gene. Mamm Genome 17: 1025–1032 doi:10.1007/s00335-006-0033-7 17019651 10.1007/s00335-006-0033-7

[20] Nam J-S, Park E, Turcotte TJ, Palencia S, Zhan X, et al (2007) Mouse R-spondin2 is required for apical ectodermal ridge maintenance in the hindlimb. Dev Biol 311: 124–135 doi:10.1016/j.ydbio.2007.08.023 17904116 10.1016/j.ydbio.2007.08.023 PMC2692258

[21] Smidt MP, Smits SM, Bouwmeester H, Hamers FPT, Van der Linden AJA, et al (2004) Early developmental failure of substantia nigra dopamine neurons in mice lacking the homeodomain gene Pitx3. Development 131: 1145–1155 doi:10.1242/dev.01022 14973278 10.1242/dev.01022

[22] Smits SM, Ponnio T, Conneely OM, Burbach JPH, Smidt MP (2003) Involvement of Nurr1 in specifying the neurotransmitter identity of ventral midbrain dopaminergic neurons. Eur J Neurosci 18: 1731–1738. 14622207 10.1046/j.1460-9568.2003.02885.x

[23] Grima B, Lamouroux A, Blanot F, Biguet NF, Mallet J (1985) Complete coding sequence of rat tyrosine hydroxylase mRNA. Proc Natl Acad Sci USA 82: 617–621. 2857492 10.1073/pnas.82.2.617 PMC397092

[24] Hamatani T, Falco G, Carter MG, Akutsu H, Stagg CA, et al (2004) Age-associated alteration of gene expression patterns in mouse oocytes. Hum Mol Genet 13: 2263–2278 doi:10.1093/hmg/ddh241 15317747 10.1093/hmg/ddh241

[25] Yang YH, Dudoit S, Luu P, Lin DM, Peng V, et al (2002) Normalization for cDNA microarray data: a robust composite method addressing single and multiple slide systematic variation. Nucleic Acids Res 30: e15. 11842121 10.1093/nar/30.4.e15 PMC100354

[26] Wu TD (2002) Large-Scale Analysis of Gene Expression Profiles. Brief Bioinform 3: 7–17 doi:10.1093/bib/3.1.7 12002225 10.1093/bib/3.1.7

[27] Zou H-L, Su C-J, Shi M, Zhao G-Y, Li Z-Y, et al (2009) Expression of the LIM-homeodomain gene Lmx1a in the postnatal mouse central nervous system. Brain Research Bulletin 78: 306–312 doi:10.1016/j.brainresbull.2008.12.001 19111912 10.1016/j.brainresbull.2008.12.001

[28] Puelles L, Rubenstein JLR (2003) Forebrain gene expression domains and the evolving prosomeric model. Trends Neurosci 26: 469–476. 12948657 10.1016/S0166-2236(03)00234-0

[29] Nakatani T, Kumai M, Mizuhara E, Minaki Y, Ono Y (2010) Lmx1a and Lmx1b cooperate with Foxa2 to coordinate the specification of dopaminergic neurons and control of floor plate cell differentiation in the developing mesencephalon. Dev Biol 339: 101–113 doi:10.1016/j.ydbio.2009.12.017 20035737 10.1016/j.ydbio.2009.12.017

[30] Hoekstra EJ, Von Oerthel L, Van der Linden AJA, Schellevis RD, Scheppink G, et al (2013) Lmx1a is an activator of Rgs4 and Grb10 and is responsible for the correct specification of rostral and medial mdDA neurons. Eur J Neurosci 37: 23–32 doi:10.1111/ejn.12022 23106268 10.1111/ejn.12022

[31] Jacobs FMJ, Smits SM, Noorlander CW, Von Oerthel L, Van der Linden AJA, et al (2007) Retinoic acid counteracts developmental defects in the substantia nigra caused by Pitx3 deficiency. Development 134: 2673–2684 doi:10.1242/dev.02865 17592014 10.1242/dev.02865

[32] Jacobs FMJ, Van der Linden AJA, Wang Y, Von Oerthel L, Sul HS, et al (2009) Identification of Dlk1, Ptpru and Klhl1 as novel Nurr1 target genes in meso-diencephalic dopamine neurons. Development 136: 2363–2373 doi:10.1242/dev.037556 19515692 10.1242/dev.037556 PMC3266485

[33] Jacobs FMJ, Veenvliet JV, Almirza WH, Hoekstra EJ, Von Oerthel L, et al (2011) Retinoic acid-dependent and -independent gene-regulatory pathways of Pitx3 in meso-diencephalic dopaminergic neurons. Development 138: 5213–5222 doi:10.1242/dev.071704 22069189 10.1242/dev.071704

[34] Sánchez-Danés A, Consiglio A, Richaud Y, Rodríguez-Pizà I, Dehay B, et al (2012) Efficient generation of A9 midbrain dopaminergic neurons by lentiviral delivery of LMX1A in human embryonic stem cells and induced pluripotent stem cells. Hum Gene Ther 23: 56–69 doi:10.1089/hum.2011.054 21877920 10.1089/hum.2011.054 PMC3472681

[35] Friling S, Andersson E, Thompson LH, Jönsson ME, Hebsgaard JB, et al (2009) Efficient production of mesencephalic dopamine neurons by Lmx1a expression in embryonic stem cells. Proc Natl Acad Sci USA 106: 7613–7618 doi:10.1073/pnas.0902396106 19383789 10.1073/pnas.0902396106 PMC2671325

[36] Barzilay R, Ben-Zur T, Bulvik S, Melamed E, Offen D (2009) Lentiviral delivery of LMX1a enhances dopaminergic phenotype in differentiated human bone marrow mesenchymal stem cells. Stem Cells Dev 18: 591–601 doi:10.1089/scd.2008.0138 19298173 10.1089/scd.2008.0138

[37] Roybon L, Hjalt T, Christophersen NS, Li J-Y, Brundin P (2008) Effects on differentiation of embryonic ventral midbrain progenitors by Lmx1a, Msx1, Ngn2, and Pitx3. J Neurosci 28: 3644–3656 doi:10.1523/JNEUROSCI.0311-08.2008 18385323 10.1523/JNEUROSCI.0311-08.2008 PMC6671084

[38] Brandt MD, Jessberger S, Steiner B, Kronenberg G, Reuter K, et al (2003) Transient calretinin expression defines early postmitotic step of neuronal differentiation in adult hippocampal neurogenesis of mice. Molecular and Cellular Neuroscience 24: 603–613 doi:10.1016/S1044-7431(03)00207-0 14664811 10.1016/s1044-7431(03)00207-0

[39] Niculescu MD, Craciunescu CN, Zeisel SH (2006) Dietary choline deficiency alters global and gene-specific DNA methylation in the developing hippocampus of mouse fetal brains. FASEB J 20: 43–49 doi:10.1096/fj.05-4707com 16394266 10.1096/fj.05-4707com PMC1635129

[40] Khalil AM, Guttman M, Huarte M, Garber M, Raj A, et al (2009) Many human large intergenic noncoding RNAs associate with chromatin-modifying complexes and affect gene expression. Proc Natl Acad Sci U S A 106: 11667–11672 doi:10.1073/pnas.0904715106 19571010 10.1073/pnas.0904715106 PMC2704857

[41] Han XH, Jin Y-R, Seto M, Yoon JK (2011) A WNT/beta-catenin signaling activator, R-spondin, plays positive regulatory roles during skeletal myogenesis. J Biol Chem 286: 10649–10659 doi:10.1074/jbc.M110.169391 21252233 10.1074/jbc.M110.169391 PMC3060516

[42] Jin Y-R, Turcotte TJ, Crocker AL, Han XH, Yoon JK (2011) The canonical Wnt signaling activator, R-spondin2, regulates craniofacial patterning and morphogenesis within the branchial arch through ectodermal-mesenchymal interaction. Dev Biol 352: 1–13 doi:10.1016/j.ydbio.2011.01.004 21237142 10.1016/j.ydbio.2011.01.004 PMC3089906

[43] Yoon JK, Lee J-S (2012) Cellular signaling and biological functions of R-spondins. Cell Signal 24: 369–377 doi:10.1016/j.cellsig.2011.09.023 21982879 10.1016/j.cellsig.2011.09.023 PMC3237830

[44] Yamada W, Nagao K, Horikoshi K, Fujikura A, Ikeda E, et al (2009) Craniofacial malformation in R-spondin2 knockout mice. Biochem Biophys Res Commun 381: 453–458 doi:10.1016/j.bbrc.2009.02.066 19233133 10.1016/j.bbrc.2009.02.066

[45] Bell SM, Schreiner CM, Wert SE, Mucenski ML, Scott WJ, et al (2008) R-spondin 2 is required for normal laryngeal-tracheal, lung and limb morphogenesis. Development 135: 1049–1058 doi:10.1242/dev.013359 18256198 10.1242/dev.013359

[46] Nam J-S, Turcotte TJ, Yoon JK (2007) Dynamic expression of R-spondin family genes in mouse development. Gene Expression Patterns 7: 306–312 doi:10.1016/j.modgep.2006.08.006 17035101 10.1016/j.modgep.2006.08.006

[47] Kim K-A, Wagle M, Tran K, Zhan X, Dixon MA, et al (2008) R-Spondin Family Members Regulate the Wnt Pathway by a Common Mechanism. Mol Biol Cell 19: 2588–2596 doi:10.1091/mbc.E08-02-0187 18400942 10.1091/mbc.E08-02-0187 PMC2397303

[48] Nam J-S, Turcotte TJ, Smith PF, Choi S, Yoon JK (2006) Mouse cristin/R-spondin family proteins are novel ligands for the Frizzled 8 and LRP6 receptors and activate beta-catenin-dependent gene expression. J Biol Chem 281: 13247–13257 doi:10.1074/jbc.M508324200 16543246 10.1074/jbc.M508324200

[49] Klauzinska M, Baljinnyam B, Raafat A, Rodriguez-Canales J, Strizzi L, et al (2012) Rspo2/Int7 regulates invasiveness and tumorigenic properties of mammary epithelial cells. Journal of Cellular Physiology 227: 1960–1971 doi:10.1002/jcp.22924 21732367 10.1002/jcp.22924 PMC6957254

[50] Kazanskaya O, Glinka A, Del Barco Barrantes I, Stannek P, Niehrs C, et al (2004) R-Spondin2 is a secreted activator of Wnt/beta-catenin signaling and is required for Xenopus myogenesis. Dev Cell 7: 525–534 doi:10.1016/j.devcel.2004.07.019 15469841 10.1016/j.devcel.2004.07.019

[51] Kim K-A, Zhao J, Andarmani S, Kakitani M, Oshima T, et al (2006) R-Spondin proteins: a novel link to beta-catenin activation. Cell Cycle 5: 23–26. 16357527 10.4161/cc.5.1.2305

[52] Megason SG, McMahon AP (2002) A mitogen gradient of dorsal midline Wnts organizes growth in the CNS. Development 129: 2087–2098. 11959819 10.1242/dev.129.9.2087

