# Supplementary figures and images for: Lmx1a Encodes a Rostral Set of Mesodiencephalic Dopaminergic Neurons Marked by the Wnt/B-Catenin Signaling Activator R-spondin 2
Source: PLoS One. 2013 Sep 16;8(9):e74049. doi: 10.1371/journal.pone.0074049 (PMC3774790; doi:10.1371/journal.pone.0074049)

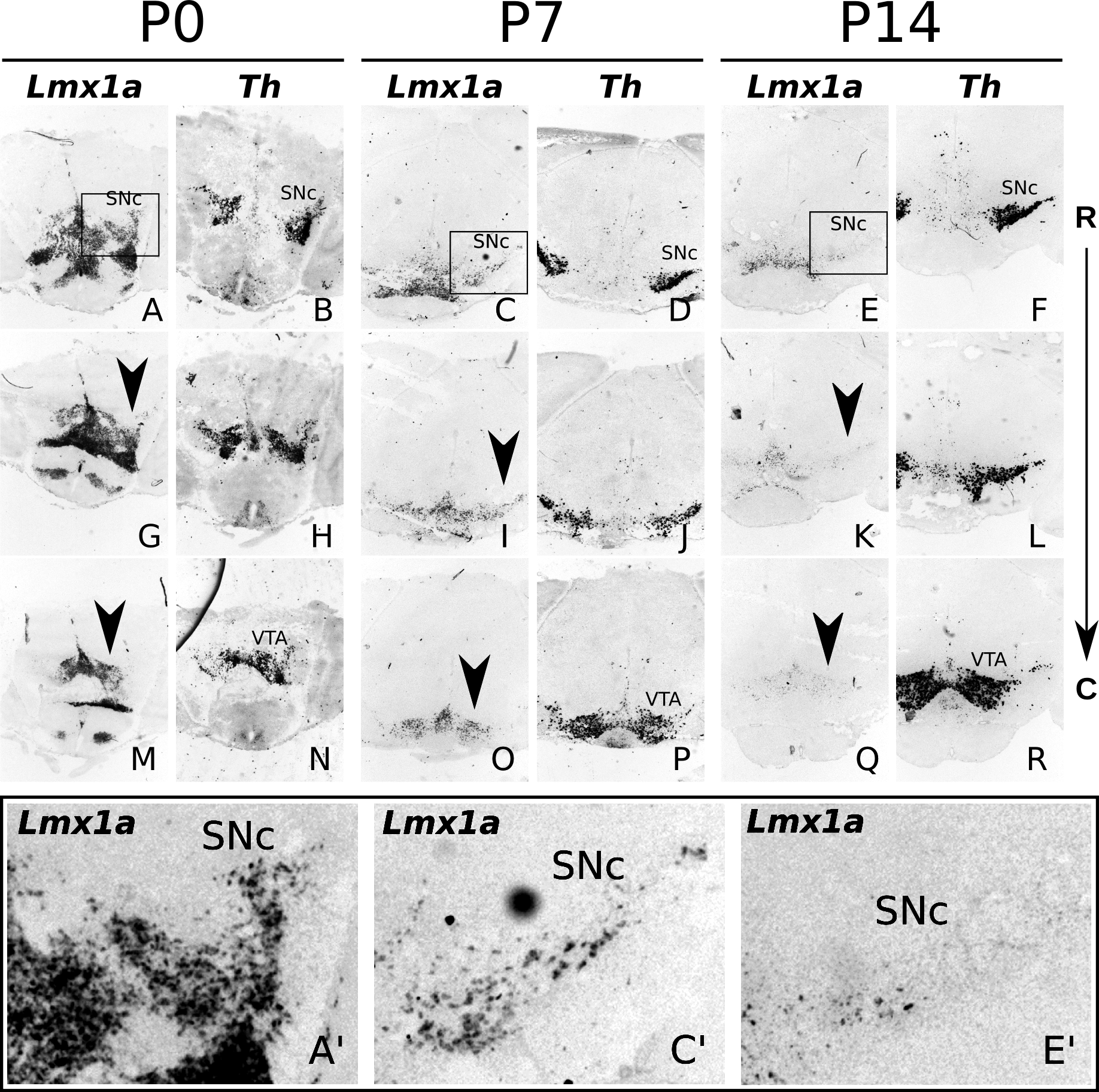

Supplement: Figure S1 — Lmx1a expression is down-regulated shortly after birth. Coronal sections of wild-type mouse tissue at P0, P7 and P14. In situ hybridization of Lmx1a and Th is shown from rostral to caudal. Th was taken along to mark the SNc and VTA and as a control for transcript levels. (A–F) A significant loss of Lmx1a expression is observed in the SNc at P14, when compared to P7 or P0, and when compared to Th. (A′,C′,E′) Lmx1a expression in the SNc in more detail, showing that only few cells remain that express Lmx1a, in low levels. (G–R) Both in caudal SNc, and more caudally, the VTA, the expression of Lmx1a is clearly diminished at P14 (arrowheads). P, postnatal day; SNc, Substantia nigra pars compacta; VTA, ventral tegmental area. (TIF) [file pone.0074049.s001.tif]

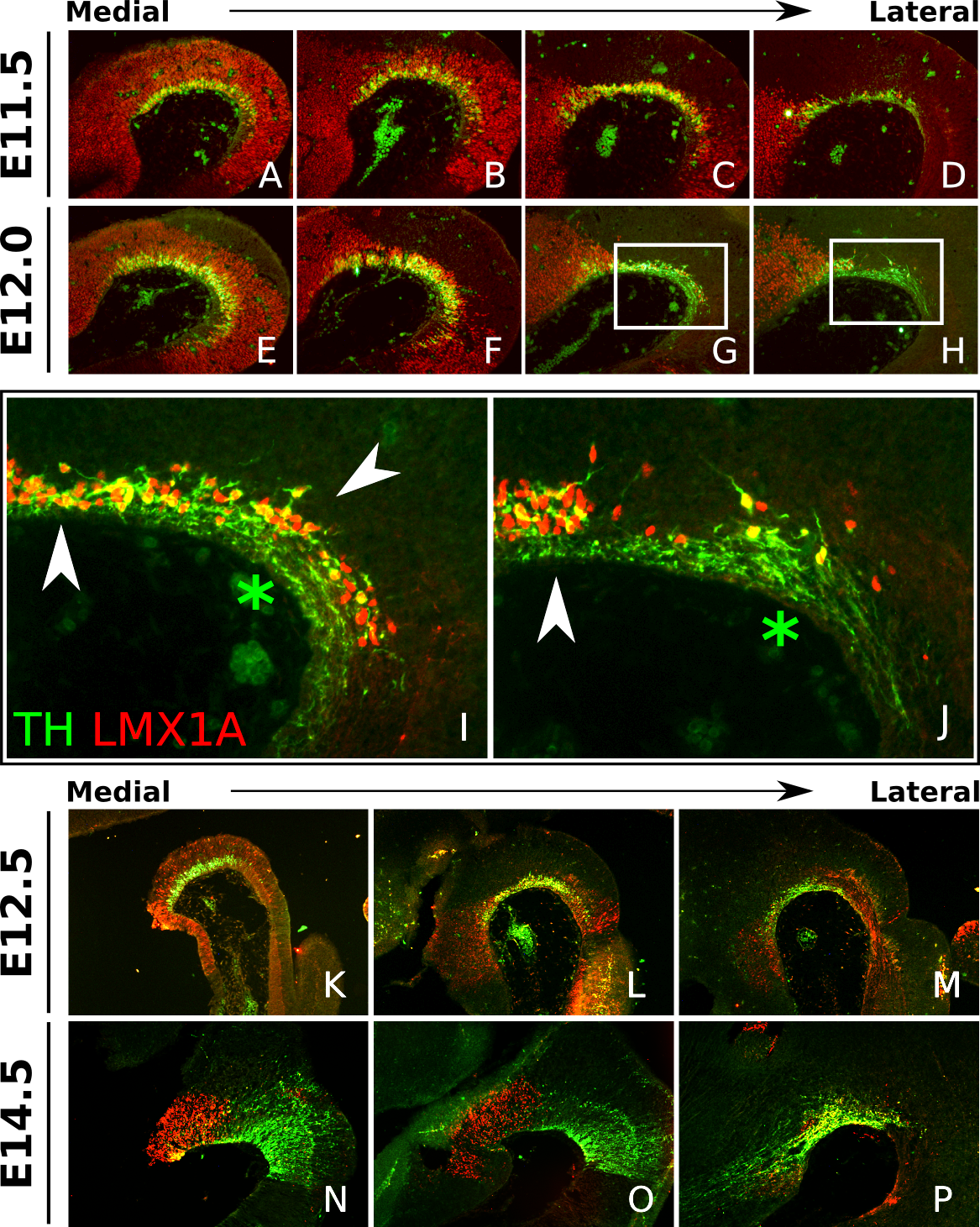

Supplement: Figure S2 — LMX1A protein expression is restricted to rostral-lateral TH expression at later developmental stages. (A–H) IHC on E11.5 (A–D) and E12.0 (E–H) sagittal wild-type mouse tissue, showing LMX1A protein (red) and TH protein (green). Medially, full protein overlap is displayed, whereas in lateral expression domains, only a subset of TH+ neurons overlaps with LMX1A. (I–J) Higher magnifications of G and H, showing the group of TH+ neurons that co-localize with LMX1A (white arrowheads), and a group of TH expressing neurons that do not express LMX1A (green asterisks). (K–M) The observed rostral-lateral overlap is more clear at E12.5, where also in the medial brain, a rostral specificity of LMX1A occurs. (N–P) At E14.5, the rostral LMX1A/TH restriction is clearly observed in medial and lateral midbrain areas. (TIF) [file pone.0074049.s002.tif]

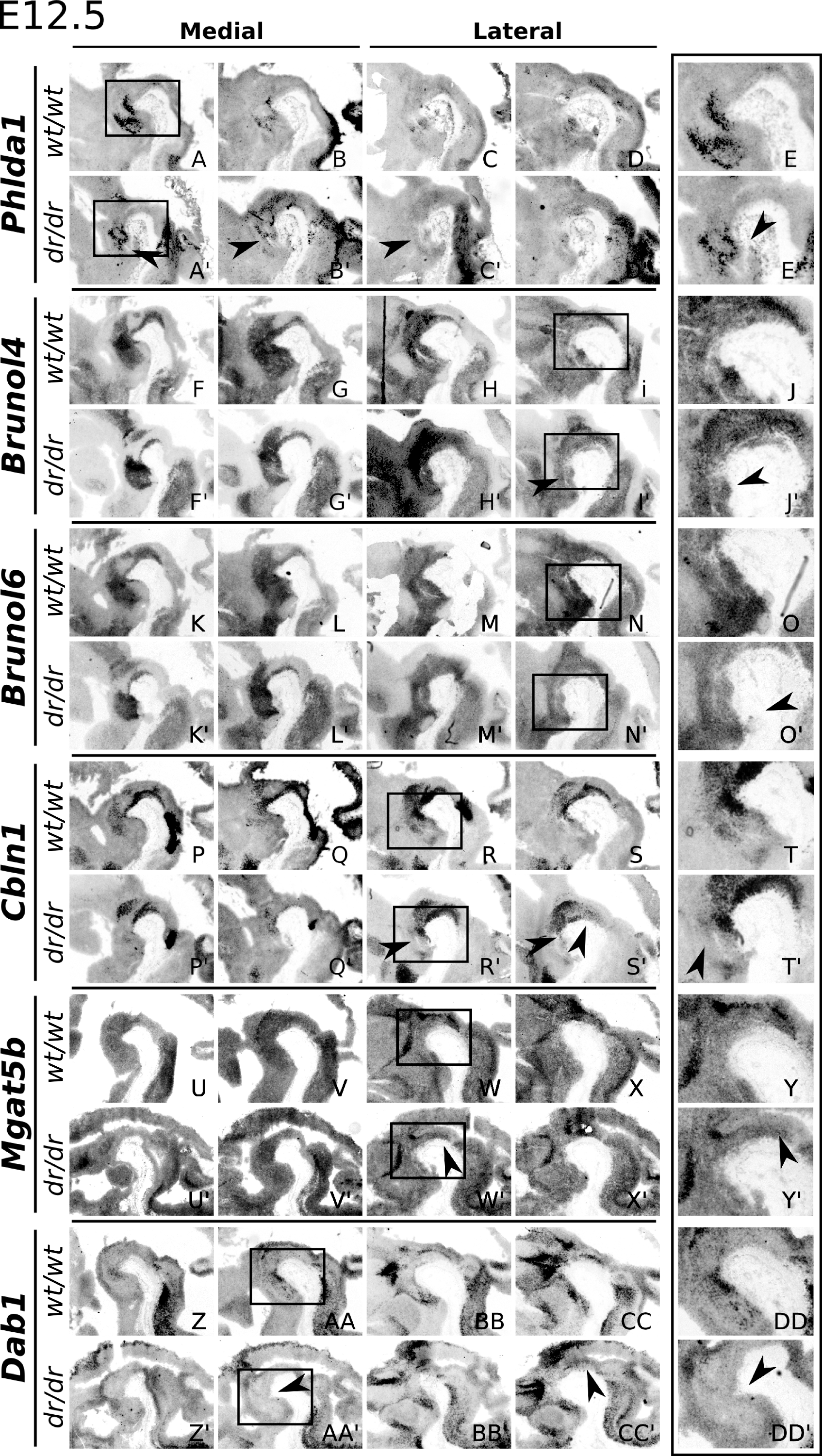

Supplement: Figure S3 — Lmx1a regulates P hlda1, Brunol4, Brunol6, Cbln1, Mgat5b and Dab1 in selective areas of the Lmx1a-expression domain. Sagittal Lmx1a control and Lmx1a-dr/dr sections, medial and lateral. On the right, higher magnifications of the boxed areas are shown. (A–E′) Phlda1 expression is slightly reduced in rostral parts of the brain (arrowheads). (F–O′) Brunol4 and Brunol6 both show a slight down-regulation in rostral-lateral domains (G,G′, and arrowheads). (P–T′) Cbln1 expression is diminished mainly in rostral areas. (U–Y′) Mgat5b transcript levels are slightly reduced in the lateral-caudal midbrain (arrowheads). (Z–DD′) Dab1 shows loss of expression in medial-rostral areas (Z′,AA′,DD′, arrowheads). Also lateral-caudal, expression is affected (CC′, arrowhead). (TIF) [file pone.0074049.s003.tif]

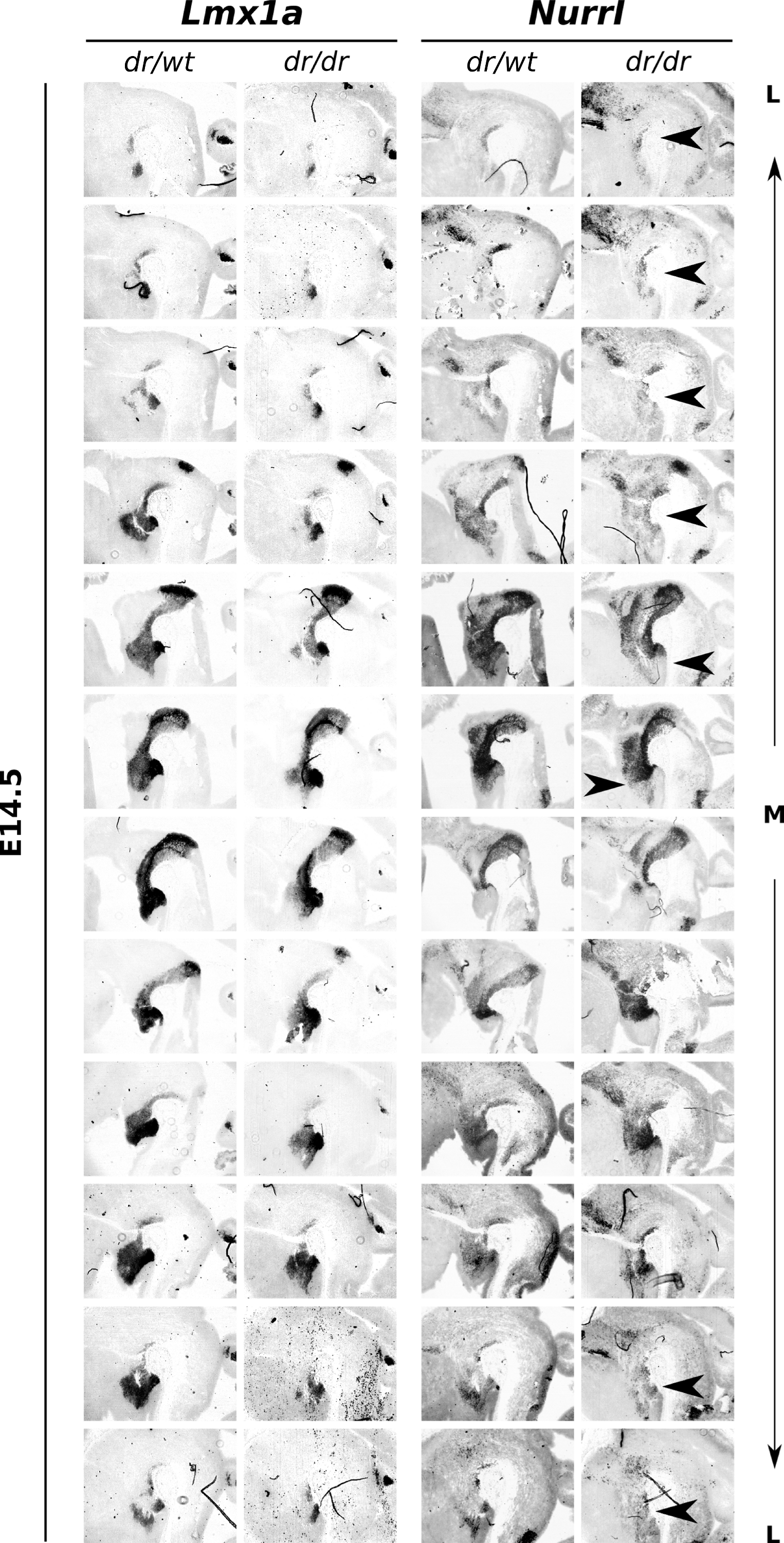

Supplement: Figure S4 — Lmx1a and NurrI expression throughout the sagittal brain at E14.5. Lmx1a expression (left columns) from lateral (L) to medial (M) to lateral brain domains, in wild-type and Lmx1a-dr/dr tissue, at E14.5. In lateral domains, Lmx1a expression is down-regulated in the Lmx1a knock-out, and in all sections throughout the brain, a rostral defect is shown. NurrI expression (right columns) was analyzed in the same set-up. For NurrI a drastically decrease in rostral and lateral expression can be observed (arrowheads). (TIF) [file pone.0074049.s004.tif]

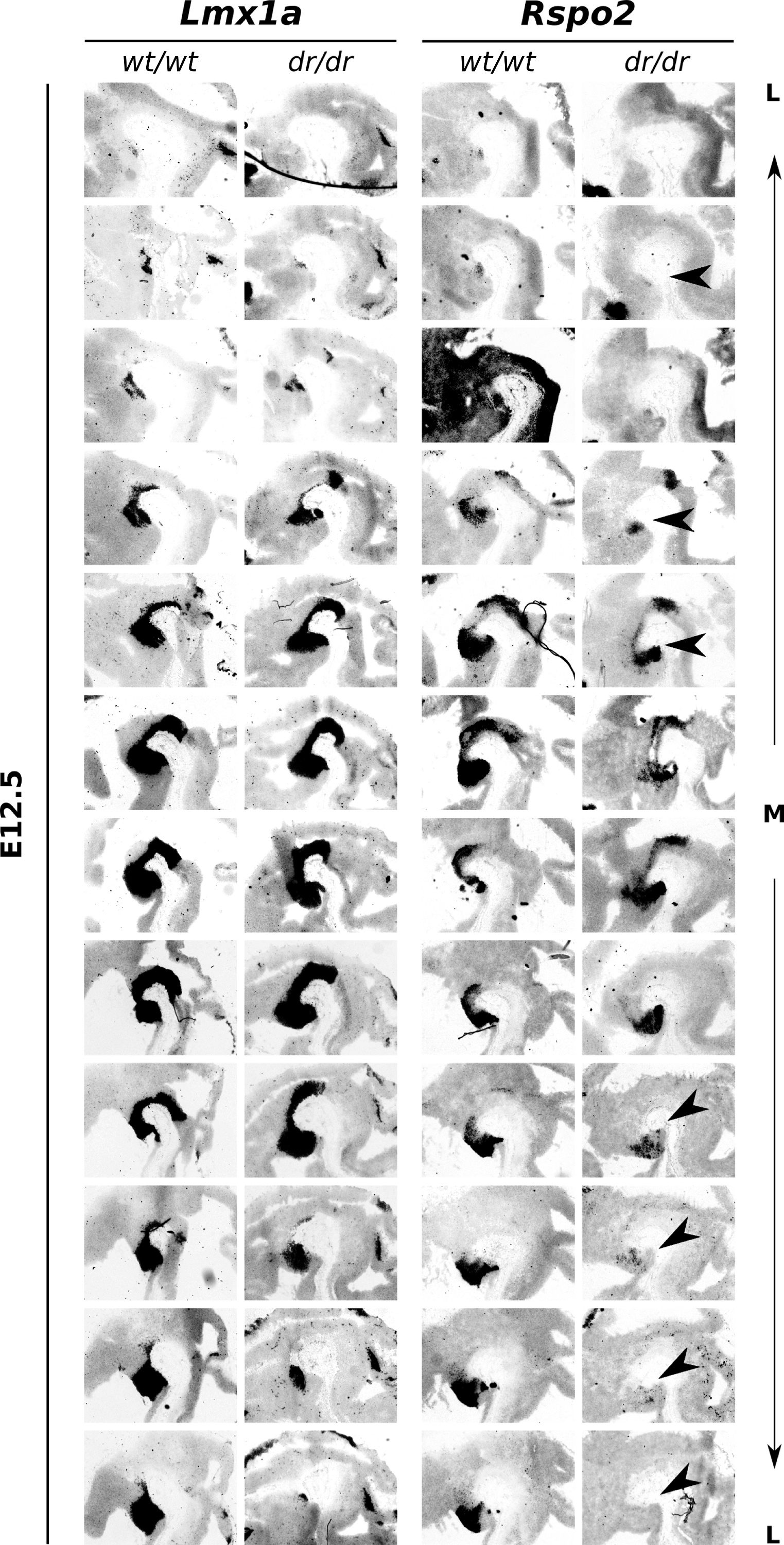

Supplement: Figure S5 — Lmx1a and Rspo2 expression throughout the sagittal brain at E12.5. Lmx1a expression (left columns) from lateral (L) to medial (M) to lateral brain domains, in wild-type and Lmx1a-dr/dr tissue, at E12.5. In lateral positions, Lmx1a expression is clearly down-regulated in the Lmx1a knock-out, and in all sections, a rostral defect can be seen. Rspo2 expression (right columns) was analyzed in the same set-up. For Rspo2 an even more drastic decrease in rostral and lateral expression can be observed (arrowheads). (TIF) [file pone.0074049.s005.tif]

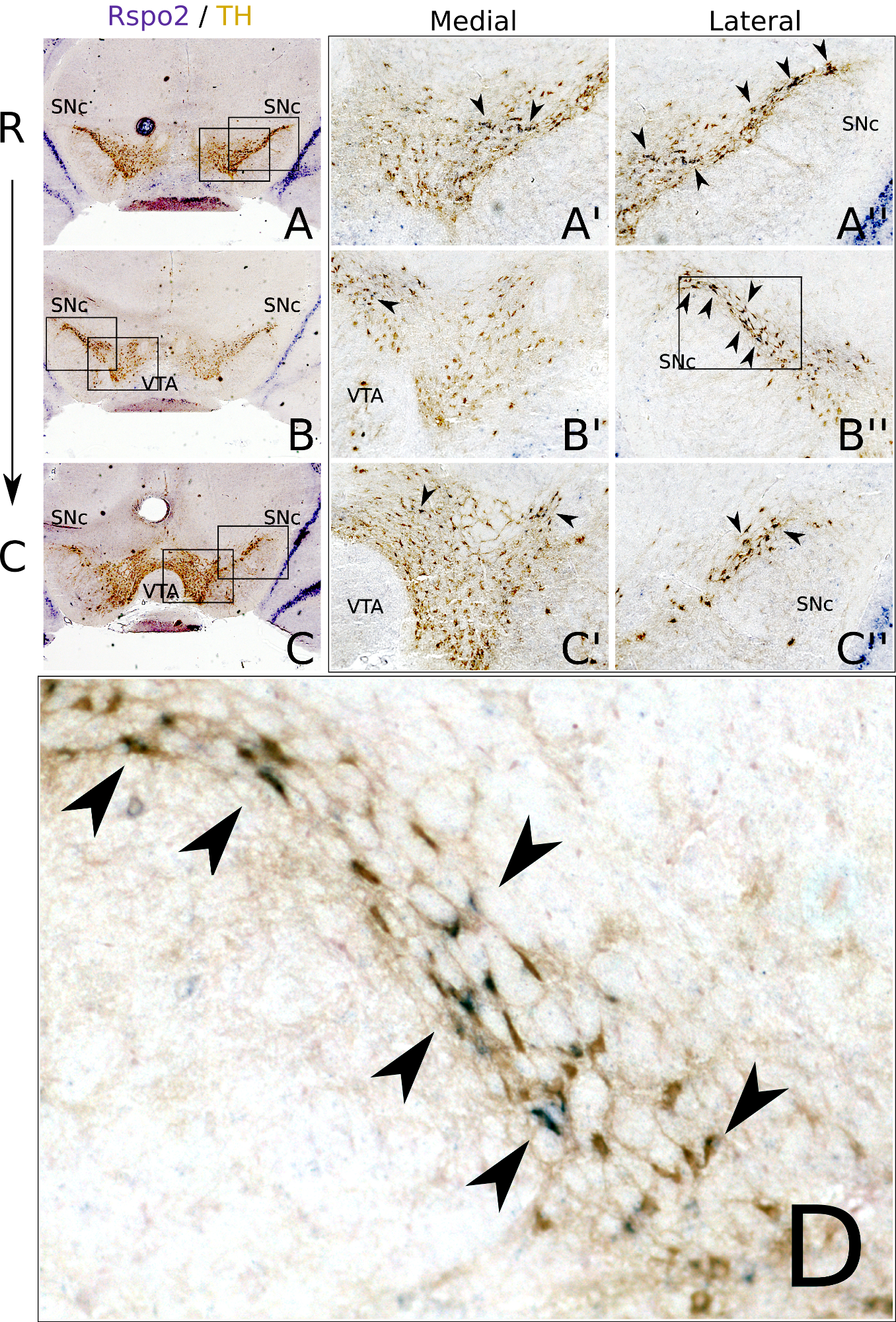

Supplement: Figure S6 — Rspo2 co-localizes with TH in adult mdDA neurons. (A–C) Combined ISH/IHC for Rspo2 (blue) and TH (brown). Most Rspo2-positive cells are found in the rostral and lateral mdDA system, in the SNc (A′′,B′′,C′′, arrowheads) and also some cells expressing Rspo2 are observed in the VTA (A′,B′,C′). All Rspo2-positive cells also express TH, indicating that Rspo2 is expressed in a subset of mdDA neurons (D). (TIF) [file pone.0074049.s006.tif]
