# Supplementary material for: Lmx1a Encodes a Rostral Set of Mesodiencephalic Dopaminergic Neurons Marked by the Wnt/B-Catenin Signaling Activator R-spondin 2
Source: PLoS One. 2013 Sep 16;8(9):e74049. doi: 10.1371/journal.pone.0074049 (PMC3774790; doi:10.1371/journal.pone.0074049)
Supplement: Table S3 — A selection of genes regulated in E12.5 Lmx1a-dr/dr mouse embryos. (PDF) [file pone.0074049.s009.pdf]

Table S3. A selection of genes regulated in E12.5 *Lmx1a-dr/dr* mouse embryos

Gene symbol	Description	Fold-change	Log2-ratio	p-value
**Genes implicated in mdDA development/function**				
Nr4a2	Nuclear receptor subfamily 4 group A member 2 (Nurr1)	0.59	-0.75	0.0020
C130021l20Rik	Putative uncharacterized protein	0,64	-0,64	0.0002
Lmx1a	LIM homeobox transcription factor 1-alpha (LMX-1.1)	0.65	-0.61	0.0000
TH	Tyrosine 3-monooxygenase (Tyrosine 3-hydroxylase)(TH)	0.68	-0.56	0.0006
Brunol6	CUG-BP- and ETR-3-like factor 6 (CELF-6)(Bruno-like protein 6)	0.70	-0.51	0.0022
Arx	Homeobox protein ARX (Aristaless-related homeobox)	0.73	-0.46	0.0018
**Transcription factors**				
Nr4a2	Nuclear receptor subfamily 4 group A member 2 (Nurr1)	0.59	-0.75	0.0020
Dtx3	Protein deltex-3 (Deltex3) (mDTX3)	0.64	-0.64	0.0002
Lmx1a	LIM homeobox transcription factor 1-alpha (LMX-1.1)	0.65	-0.61	0.0000
Pa2g4	Proliferation-associated protein 2G4	0.68	-0.56	0.0012
Thra	Thyroid hormone receptor alpha (Nr1a1)	0.68	-0.55	0.0002
Pitx2	Pituitary homeobox 2 (Paired-like HD transcription factor 2)	0.69	-0.53	0.0006
Zzef1	Zinc finger ZZ-type and EF-hand domain-containing protein 1	0.70	-0.50	0.0014
Bat2	Large proline-rich protein BAT2 (HLA-B-associated transcript 2)	0.71	-0.50	0.0020
Arx	Homeobox protein ARX (Aristaless-related homeobox)	0.73	-0.46	0.0018
Casz1	Zinc finger protein castor homolog 1 (Castor-related protein)	0.74	-0.43	0.0066
Med25	Mediator of RNA polymerase II transcription subunit 25	0.75	-0.41	0.0064
Meis3	Homeobox protein Meis3 (Meis1-related protein 2)	0.76	-0.40	0.0024
Sf1	Splicing factor 1 (Zinc finger protein 162)	0.76	-0.39	0.0044
Cnot4	CCR4-NOT transcription complex subunit 4	1.33	0.41	0.0034
Lhx9	LIM/homeobox protein Lhx9 (LIM homeobox protein 9)	1.34	0.42	0.0438
Pou4f1	POU domain, class 4, transcription factor 1 (Brn-3A)(Brn-3.0).	1,44	0.52	0.0044
Rnf152	RING finger protein 152	1.44	0.53	0.0060
Pbx1	Pre-B-cell leukemia transcription factor 1 (Homeobox protein PBX1)	1,77	0.82	0.0000
Usf1	Upstream stimulatory factor 1 (Major late transcription factor 1)	1.82	0.86	0.0012
**Homeodomain genes**				
Lmx1a	LIM homeobox transcription factor 1-alpha (LMX-1.1)	0.65	-0.61	0.0000
Pitx2	Pituitary homeobox 2 (Paired-like HD transcription factor 2)	0.69	-0.53	0.0006
Arx	Homeobox protein ARX (Aristaless-related homeobox)	0.73	-0.46	0.0018
Meis3	Homeobox protein Meis3 (Meis1-related protein 2)	0.76	-0.40	0.0024
Zfhx2	Zinc finger homeobox 2	0.78	-0.36	0.0132
Lhx9	LIM/homeobox protein Lhx9 (LIM homeobox protein 9)	1.34	0.42	0.0438
Pou4f1	POU domain, class 4, transcription factor 1 (Brn-3A)(Brn-3.0).	1,44	0.52	0.0044
Pbx1	Pre-B-cell leukemia transcription factor 1 (Homeobox protein PBX1)	1,77	0.82	0.0000
**Axon guidance**				
Vangl2	Vang-like protein 2 (Van Gogh-like protein 2)	0.48	-1.06	0.0012
Sema4g	Semaphorin-4G Precursor	0.74	-0.43	0.0122
**Migration**				
Dab1	Disabled homolog 1	0.79	-0.34	0.0188
